# Dynamical Mechanism of Hyperpolarization-Activated Non-specific Cation Current Induced Resonance and Spike-Timing Precision in a Neuronal Model

**DOI:** 10.3389/fncel.2018.00062

**Published:** 2018-03-08

**Authors:** Zhiguo Zhao, Li Li, Huaguang Gu

**Affiliations:** ^1^School of Aerospace Engineering and Applied Mechanics, Tongji University, Shanghai, China; ^2^School of Basic Science, Henan Institute of Technology, Xinxiang, China

**Keywords:** hyperpolarization-activated current, post-inhibitory rebound, subthreshold resonance, spike-timing precision, bifurcations, temporal coding

## Abstract

Hyperpolarization-activated cyclic nucleotide-gated cation current (*I*_*h*_) plays important roles in the achievement of many physiological/pathological functions in the nervous system by modulating the electrophysiological activities, such as the rebound (spike) to hyperpolarization stimulations, subthreshold membrane resonance to sinusoidal currents, and spike-timing precision to stochastic factors. In the present paper, with increasing *g*_*h*_ (conductance of *I*_*h*_), the rebound (spike) and subthreshold resonance appear and become stronger, and the variability of the interspike intervals (ISIs) becomes lower, i.e., the enhancement of spike-timing precision, which are simulated in a conductance-based theoretical model and well explained by the nonlinear concept of bifurcation. With increasing *g*_*h*_, the stable node to stable focus, to coexistence behavior, and to firing via the codimension-1 bifurcations (Hopf bifurcation, saddle-node bifurcation, saddle-node bifurcations on an invariant circle, and saddle homoclinic orbit) and codimension-2 bifurcations such as Bogdanov-Takens (BT) point related to the transition between saddle-node and Hopf bifurcations, are acquired with 1- and 2-parameter bifurcation analysis. The decrease of variability of ISIs with increasing *g*_*h*_ is induced by the fast decrease of the standard deviation of ISIs, which is related to the increase of the capacity of resisting noisy disturbance due to the firing becomes far away from the bifurcation point. The enhancement of the rebound (spike) with increasing *g*_*h*_ builds up a relationship to the decrease of the capacity of resisting disturbance like the hyperpolarization stimulus as the resting state approaches the bifurcation point. The “typical”-resonance and non-resonance appear in the parameter region of the stable focus and node far away from the bifurcation points, respectively. The complex or “strange” dynamics, such as the “weak”-resonance for the stable node near the transition point between the stable node and focus and the non-resonance for the stable focus close to the codimension-1 and −2 bifurcation points, are discussed.

## Introduction

The hyperpolarization-activated current (*I*_*h*_) mediated by hyperpolarization-activated cyclic nucleotide-gated (HCN) cation ion channels have been identified to contribute to many physiological and pathological functions in the nervous systems, such as neocortical spiny interneurons, cerebellar Purkinje cells, rod photoreceptors, hippocampal pyramidal neurons, inspiratory neurons, motor neurons, and thalamic relay neurons (Pape, 1996; Wahl-Schott and Biel, 2009; He et al., 2014; DiFrancesco and DiFrancesco, 2015). The functions associated with *I*_*h*_ include learning and memory, sleep and wakefulness, spatial reference and navigation, and circadian control (Pape, 1996; Wahl-Schott and Biel, 2009; Buzsáki and Moser, 2013; He et al., 2014; DiFrancesco and DiFrancesco, 2015). The dysregulation of HCN channels in the nervous system may lead to pathological conditions such as epilepsy (DiFrancesco et al., 2011; Reid et al., 2012), neuropathic pain (Emery et al., 2012; Schnorr et al., 2014), and Parkinsonian disease (Good et al., 2011; Masi et al., 2013). These physiological or pathological functions related to *I*_*h*_ are achieved by modulating the electrophysiological activities, such as the resting membrane potential, neuronal excitability, rhythmic activity, dendritic integration, and synaptic transmission (He et al., 2014; DiFrancesco and DiFrancesco, 2015).

The marked characteristics of the electrophysiological activities induced by *I*_*h*_ are the voltage sag and post-inhibitory rebound (spike) to hyperpolarization stimulus, due to *I*_*h*_ is a slow inward current activated by the hyperpolarization stimulation rather than depolarization stimulation (Ascoli et al., 2010; Engbers et al., 2011; Gastrein et al., 2011; Pavlov et al., 2011; Gonzalez et al., 2015). Furthermore, the experimental studies found that *I*_*h*_ can induce subthreshold membrane resonance and enhance spike-timing precision characterized by the standard deviation (*STD*) or coefficient variation (*CV*) of the interspike intervals (ISIs) (Zemankovics et al., 2010; Gastrein et al., 2011; Pavlov et al., 2011; Borel et al., 2013; Gonzalez et al., 2015). Resonance means that impedance profile of a neuron to external stimulus reaches a maximal value at a certain value corresponding to an intrinsic frequency preference (Rotstein, 2015; Vazifehkhah et al., 2015), which is an important factor related to the rhythmic oscillations (Gasparini and DiFrancesco, 1997; Hutcheon and Yarom, 2000; Giocomo et al., 2007; Moca et al., 2014; Gonzalez et al., 2015). For example, hippocampal pyramidal neurons (Hu et al., 2002; Zemankovics et al., 2010; Gastrein et al., 2011; Borel et al., 2013) exhibit subthreshold resonance to the injection of sinusoidal current, and their resonant properties contribute to the emergence of Theta oscillations (Zemankovics et al., 2010; Stark et al., 2013). Neurons in the medial superior olive and lateral superior olive exhibit different subthreshold resonance properties which influence the efficient coding of auditory spatial cues (Remme et al., 2014). Robustness of the spike-timing to physiological noise is an essential factor for neural coding (Dayan and Abbott, 2001; Butts et al., 2007). For example, precise spiking of the lateral geniculate nucleus relay neurons (Reinagel and Reid, 2002) and many other visual neurons (Buracas et al., 1998; Masuda and Aihara, 2003) carry rich information of time-varying visual stimuli such as a strong diffuse flicker. Auditory neurons in the auditory brainstem encode information based on the timing of the individual spikes (Tzounopoulos and Kraus, 2009). Precise spike-timing of neurons also influences the synchrony of oscillation (Ratté et al., 2013). Hippocampal interneurons exhibit precise firing when Theta and Gamma oscillations appear in the hippocampus and neocortex (Hájos et al., 2004; Somogyi and Klausberger, 2005; Klausberger et al., 2006; Vida et al., 2006).

In addition to these experimental studies, the roles of *I*_*h*_ on the modulation of the neuronal electrophysiological activities, such as the membrane subthreshold resonance and spike-timing precision, have also been simulated in the theoretical models. The influences of the types of neurons, the different resonant currents, and the factors of *I*_*h*_ on the subthreshold resonance have been investigated in the theoretical models (Zemankovics et al., 2010; Rotstein and Nadim, 2014; Vazifehkhah et al., 2015; Fox et al., 2017; Pena et al., 2017). For instance, some researchers suggested that the cell-type (pyramidal cells and interneurons) specificity of the impedance or resonance profiles in the hippocampal CA1 neurons maybe explained by the properties of *I*_*h*_ combined with the passive membrane characteristics in a conductance-based theoretical model (Zemankovics et al., 2010). Investigations on the subthreshold resonance in theoretical models of the Pyloric Dilator neurons of the crab pyloric network found that *I*_*h*_ and calcium current are the dominant factors to induce the resonant properties at hyperpolarized and depolarized potentials, respectively (Vazifehkhah et al., 2015; Fox et al., 2017). *I*_*h*_ and slow potassium current were identified to play different roles in the generation of resonance (Rotstein and Nadim, 2014). The interplay between the activation kinetics and the derivative conductance of *I*_*h*_ determines the resonant frequency (Pena et al., 2017). The effect of the inputs including the synaptic input, neuron type, and intrinsic properties of neuron on the spike-timing precision related to *I*_*h*_ have been studied in the theoretical models. For example, in a simulation study, the distributed synaptic plasticity at the cerebellum input stage can regulate spike-timing on the millisecond scale (Garrido et al., 2013). It was identified that the dendritic-targeting interneurons control the spike-timing of the hippocampal CA1 pyramidal neuron via the activation of *I*_*h*_ (Park and Kwag, 2012). *I*_*h*_ in the pyramidal cells, but not in the O-LM neurons, plays an important role in the timing of spike generation, and thus the synchronization of the pyramidal cells (Orbán et al., 2006).

Compared with many experimental and simulation results (Zemankovics et al., 2010; Gastrein et al., 2011; Pavlov et al., 2011) of the subthreshold resonance and precise spike-timing induced by *I*_*h*_, there are much less theoretical investigations (Rotstein, 2015; Vazifehkhah et al., 2015) to explain the corresponding dynamical mechanism. It is well known that neuron is a nonlinear dynamical system which can be described by the differential equations, and bifurcation analysis has been identified as an effective method to identify the dynamical mechanism of the electrophysiological properties of neurons. The transition from the resting state to firing and the transition between different firing patterns can be described by the concept of bifurcation (Rinzel, 1998; Izhikevich, 2000, 2007; Tsumoto et al., 2006; Tsuji et al., 2007; Franci et al., 2012; Chen et al., 2013; Drion et al., 2015; Morozova et al., 2016; Zhao et al., 2016; Zhao and Gu, 2017). The resting states and firing patterns near the different kinds of bifurcation points exhibit different dynamics. For example, both subcritical Hopf bifurcation and saddle-node (SN) bifurcation/saddle-node on invariant circle (SNIC) bifurcation are used to characterize the transitions from the resting state to firing as the bifurcation parameter is changed. However, the resting state when perturbed and the firing pattern exhibit a fixed frequency for the subcritical Hopf bifurcation and no fixed frequency for the SN/SNIC bifurcation. The noise induced stochastic firing near the SN/SNIC bifurcation exhibits a large *CV* in ISIs and near the Hopf bifurcation manifests a low *CV*, which were used to distinguish the cortical spike trains (Gutkin and Ermentrout, 1998; Tateno et al., 2004). Although the Hopf bifurcation and SN/SNIC bifurcation have been simulated in a theoretical model to describe the activity of thalamocortical neuron, the relationships between these bifurcations and the subthreshold resonances or precise spiking-timing induced by *I*_*h*_ have not been built (Hindmarsh and Rose, 1994; Amarillo et al., 2015). The linear amplitude and phase resonances between two stable equilibrium, the focus and node, were compared in a linear model to describe the activity of an electrochemical cell (Rotstein, 2013; Rotstein and Nadim, 2014). As expected by our common knowledge, resonance should appear in the parameter region of the focus and non-resonance appears in the parameter region of the node. However, such an expectation was challenged by the complex or “strange” dynamical behaviors appearing in a narrow parameter region near the special equilibrium point with double zero eigenvalues. Within the narrow parameter region, the stable focus can exhibit non-resonance and the stable node exhibits resonance. In addition, the chaotic behaviors and period-doubling bifurcation cascades, which are induced by *I*_*h*_, have been simulated in a conductance-based neuronal bursting model to describe the activity of cold thermoreceptors (Xu et al., 2017).

In the present paper, firstly, voltage sag/rebound (spike), resonance, and precise spike-timing induced by *I*_*h*_, are simulated in a theoretical model and closely match the previous experimental observations. With increasing the conductance of *I*_*h*_, *g*_*h*_, the rebound (spike) and resonance become strong, and the *CV* of ISIs decrease, which means the enhancement of the spike-timing precision. Secondly, one-parameter bifurcation analysis and two-parameter bifurcation analysis are performed to the theoretical model. Four stable behaviors, the stable node, the stable focus, the coexistence of the resting state and firing, and the firing, locate from bottom to top in (*I*_*app*_, *g*_*h*_) plane. *I*_*app*_ is the applied current. Codimension-1 bifurcations such as Hopf, saddle-node, and saddle homoclinic orbit, and codimension-2 bifurcations such as Bogdanov-Takens (BT) related to the transition between the saddle-node and Hopf bifurcations, are acquired. Last, with help of the bifurcations, the dynamical mechanisms for *I*_*h*_ induced resonance, rebound (spike), and precise spike-timing are provided. Increasing *g*_*h*_ for the firing behavior means that it becomes far away from the bifurcation point, therefore, the capacity of resisting noisy disturbance becomes weak, which leads to the fast decrease of the standard deviation of ISIs and the decrease of *CV*. Increasing *g*_*h*_ for the resting state means that it approaches the bifurcation point, the enhancement of the rebound (spike) corresponds to the decrease of the capacity of resisting disturbance like the hyperpolarization stimulus. In addition, the “typical”-resonance and non-resonance appear in the parameter region of the stable focus and stable node far away from the bifurcation points, respectively. The “weak”-resonance appears in the parameter region of stable node near the transition point between the stable node and focus, and the non-resonance appears in the parameter region of the stable focus close to the codimension-1 and −2 bifurcation points, which are very complex or “strange”.

## Materials and methods

### A hippocampal GABAergic neurons model with *I*_*h*_

To study the influence of *I*_*h*_ on cellular electrophysiological properties, a conductance-based biophysical model of Hippocampal GABAergic neuron (Wang, 2002) is used and is described as follows,

(1) $$\begin{array}{l} C\frac{dV}{dt}=-g_{Na}m_{\infty }^{3}h\left(V-E_{Na}\right)-g_{K}n^{4}\left(V-E_{K}\right)-g_{h}H\left(V-E_{h}\right)\\ \;-g_{L}\left(V_{L}-E_{L}\right)+I_{app}, \end{array}$$

(2) $$\begin{array}{r} \frac{dh}{dt}=\phi \left(\alpha_{h}\left(1-h\right)-\beta_{h}h\right), \end{array}$$

(3) $$\begin{array}{r} \frac{dn}{dt}=\phi \left(\alpha_{n}\left(1-n\right)-\beta_{n}n\right), \end{array}$$

(4) $$\begin{array}{r} \frac{dH}{dt}=\left(H_{\infty }\left(V\right)-H\right)/\tau_{H}\left(V\right). \end{array}$$

where *C* is the membrane capacitance, and *V* is the membrane potential. *g*_*Na*_, *g*_*K*_, and *g*_*L*_ are the maximum conductances of the sodium current, potassium current, and leakage current, respectively. *E*_*Na*_, *E*_*K*_, and *E*_*L*_ are the reversal potentials of the sodium current, potassium current, and leakage current, respectively. *g*_*h*_ is the maximal conductance of *I*_*h*_ and *E*_*h*_ is the reversal potential of *I*_*h*_. *I*_*app*_ is the applied current. *m*_∞_ = α_*m*_/(α_*m*_+β_*m*_) is the steady-state function of activation variable of the sodium current. *h*, *n*, and *H* are the inactivation variables of the sodium current, activation variable of the potassium current, and activation variable of *I*_*h*_, respectively. The related expressions are as follows, α_*m*_(*V*) = −0.1(*V* + 35)/(exp(−0.1(*V* + 35)) − 1), β_*m*_(*V*) = 4exp(−(*V* + 60)/18), α_*h*_(*V*) = 0.07exp(−(*V* + 58)/20), β_*h*_(*V*) = 1/(exp(−0.1(*V* + 28)) + 1), α_*n*_(*V*) = −0.01(*V* + 34)/(exp(−0.1(*V* + 34))−1), β_*n*_(*V*) = 0.125exp(−(*V* + 44)/80), *H*_∞_(*V*) = 1/(1 + exp(*V* + 80)/10) and τ_*H*_(*V*) = 200/(exp((*V* + 70)/20) + exp(−(*V* + 70)/20)) + 5.

The parameters values are taken as *C* = 1 F/cm^2^, *g*_*Na*_ = 35 mS/cm^2^, *E*_*Na*_ = 55 mV, *g*_*K*_ = 9 mS/cm^2^, *E*_*K*_ = −90 mV, *g*_*L*_ = 0.1 mS/cm^2^, *E*_*L*_ = −65 mV, *E*_*h*_ = −30mV, and ϕ = 5.

In the experimental investigations (Gastrein et al., 2011), *I*_*h*_ was blocked and *I*_*app*_ was adjusted. In the present paper, the conductance of *I*_*h*_, *g*_*h*_, and *I*_*app*_ are chosen as the control parameters, and the equations of the theoretical model were integrated with the Euler method with a time step of 0.001 ms.

### Resonance

The impedance amplitude profile method is conventionally employed to investigate the resonance property of a neuron (Hu et al., 2002; Zemankovics et al., 2010; Engbers et al., 2011; Gastrein et al., 2011; Borel et al., 2013; Gonzalez et al., 2015; Rotstein, 2015; Vazifehkhah et al., 2015). A subthreshold “ZAP” current, which is sinusoidal current with constant amplitude and linearly increasing frequency, is injected into a neuron at the resting state, and the membrane voltage response (*V*) of the neuron is recorded. The “ZAP” current is described as

(5) $$\begin{array}{r} I_{ZAP}=I_{stim}\mathrm{Sin}\left(2\pi f\left(t\right)t\right), \end{array}$$

where $I_{stim}=0.01\mu \text{A}/\text{c}{\text{m}}^{2}$ and the time-depended frequency *f*(*t*) linearly increases from *f*_min_ = 0 to *f*_max_ = 20 Hz in the duration *T* = 20 s and is expressed as follows,

(6) $$\begin{array}{r} f\left(t\right)=f_{\min}+\left(f_{\max}-f_{\min}\right)\frac{t}{T}. \end{array}$$

The impedance profile can be obtained by calculating the ratio of the fast Fourier transforms (FFT) of the membrane voltage response to the FFT of the input “ZAP” current and is described by

(7) $$\begin{array}{r} Z\left(f\right)=\frac{FFT\left(V\right)}{FFT\left(ZAP\right)}. \end{array}$$

where *Z*(*f*) is the impedance profile and *f* is the frequency. *Z*(*f*) is a complex quantity and its magnitude |Z(f)|=(|ZRe(f)|)2+(|ZIm(f)|)2 is used to quantify the strength of the subthreshold resonance. If |*Z*(*f*)| manifests a marked maximal peak at a certain frequency *f*, the resonance appears in the neuron. If |*Z*(*f*)| does not exhibit a marked maximal peak, no resonance appears in the neuron.

### Spike-timing precision

Noise is ubiquitous in the nervous system (Faisal et al., 2008) and can disturb the spike-timing precision. To study the influence of noise on the spike-timing of a neuron, the white noise to simulate the current fluctuations *I*_*noise*_ is added to Equation and is described as follows,

(8) $$\begin{array}{r} I_{noise}=DdW\left(t\right). \end{array}$$

where *dW*(*t*) is a Gaussian white noise and *D* is the noise intensity.

For a neuron with resting state, the temporal precision of the first spike was analyzed when firing was elicited by a ramp of current that can induce membrane potential depolarization (Gastrein et al., 2011). Different ramp rates of the current induce different rates of membrane potential depolarization. For each ramp rate, multiple trials of the firings are acquired and the *STD* of the timing of the first spike for different trials is calculated. The faster the current ramp/membrane potential depolarization rate, the more precise the timing of the first spike.

**Figure 1:**
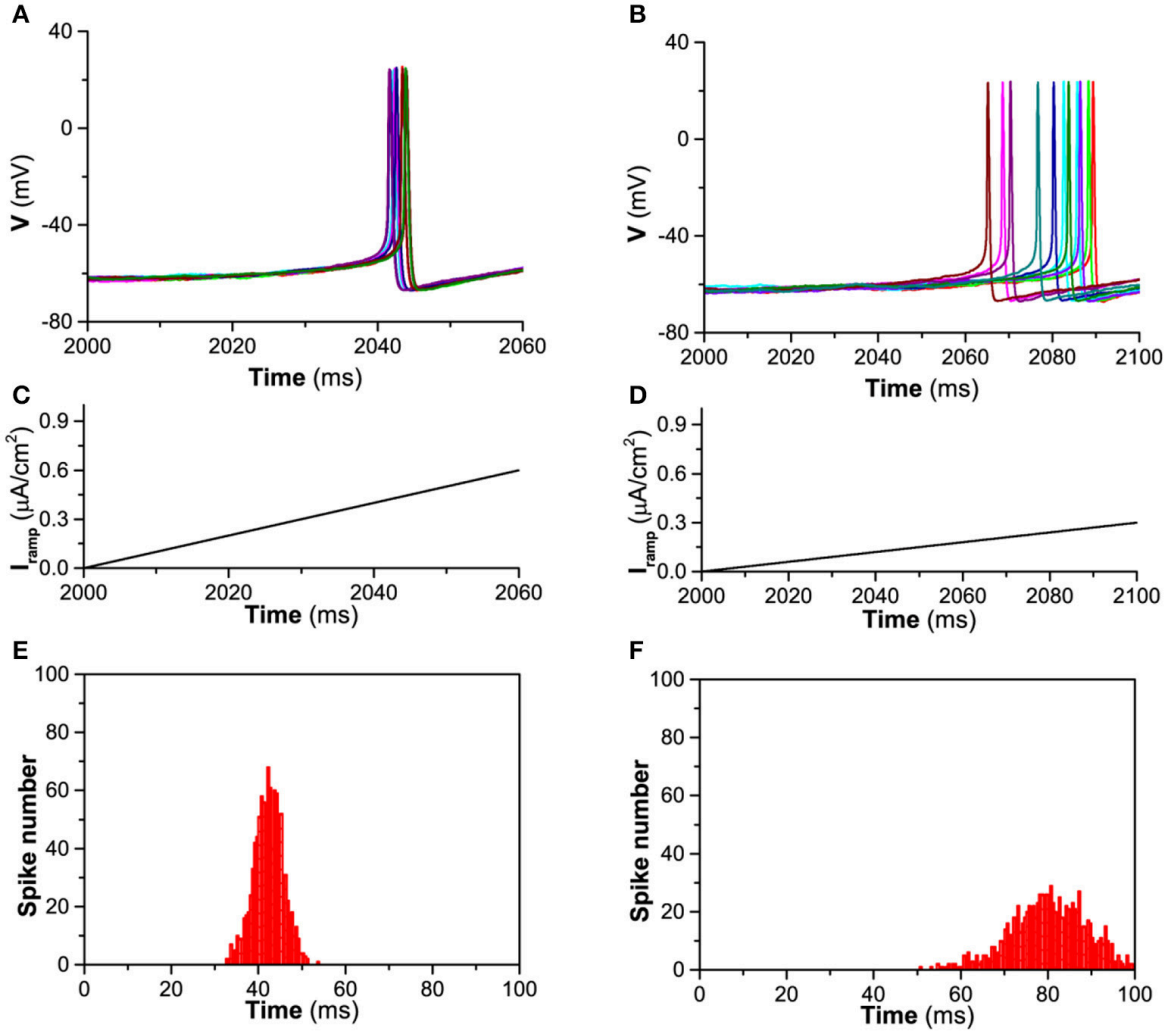
Timing precision of the first spike of firing induced by the ramp current when *D* = 0.2μA/cm^2^, *I*_*app*_ = 0 μA/cm^2^, and *g*_*h*_ = 0.02 mS/cm^2^. **(A)** The first spike of firing induced by current with fast ramp rate (12 trials); **(B)** The first spike of firing induced by current with slow ramp rate (12 trials); **(C)** Current with fast ramp rate 1 μA/100 ms; **(D)** Current with slow ramp rate 0.3 μA/100 ms; **(E)** Number of the timing of the first spike induced by current with fast ramp rate (1000 trials); **(F)** Number of the timing of the first spike induced by current with slow ramp rate (1,000 trials).

A representative for the timing of the first spike induced by the ramp current is shown in Figure 1. When *D* = 0.2μA/cm^2^, *I*_*app*_ = 0μA/cm^2^, and *g*_*h*_ = 0.02mS/cm^2^, the behavior of the theoretical model is resting state. When the currents with fast (1 μA/100 ms, Figure 1C) and slow (0.3 μA/100 ms, Figure 1D) ramp rates are applied, 12 trials of the first spike of the firings induced by the ramp currents are shown in Figures 1A,B, respectively. The *STD* of jitter timing of the first spike is 3.35 ms (mean value is 42.31 ms) for the fast ramp rate and is 8.63 ms (mean value is 80.29 ms) for the slow ramp rate. The corresponding statistics histogram calculated from 1,000 trials for the fast ramp rate is shown in Figure 1E and for the slow ramp rate in Figure 1F. The results show that the faster the current ramp, the more precise the timing of the first spike, which is consistent with the experimental observations on the stratum oriens interneurons (Gastrein et al., 2011).

For a neuron with firing, the *CV* of ISIs, which is the ratio of the standard deviation (*STD*) of ISIs to the mean value of ISIs, is used to estimate the spike-timing precision (Bacci and Huguenard, 2006; Gastrein et al., 2011; Pavlov et al., 2011), which can reflect the spike regularity. The lower the *CV* value, the more regular the spike trains, i.e., the more precise the spike-timing. In the present paper, *CV* of ISIs for firing pattern are mainly investigated and *N* = 2000 ISIs are used to calculate *CV*.

### Bifurcation analysis

In the previous experimental investigations (Gastrein et al., 2011), the resting state was considered when *I*_*h*_ induced rebound (spike) or subthreshold resonance was studied and the firing behavior was considered when *I*_*h*_ induced spike-timing precision was investigated. Therefore, the transition from the resting state to firing and the dependence of the transition on *g*_*h*_ and *I*_*app*_ is very important. The concept of nonlinear dynamics, bifurcation, describes the dynamical mechanism and presents theoretical interpretation of the transition from the resting state to firing. The transition from the resting state to the firing corresponds to the bifurcation from the stable equilibrium point to the limit cycle. The resting state corresponds to stable equilibrium point and the firing corresponds to the stable limit cycle.

The eigenvalues of the linearization of Equations. (1–4) determine the stability of the equilibrium corresponding to the resting state. There are major three types of equilibrium (Kuznetsov, 1995; Izhikevich, 2000, 2007): node, saddle, and focus. The eigenvalues of node are real and have the same sign. The eigenvalues of saddle are real and have different signs. The focus exhibits complex-conjugate eigenvalues. The resting state corresponds to a stable node with negative eigenvalues or a stable focus with eigenvalues with negative real part. When perturbed, the dynamical behavior of a focus exhibits a damped oscillation with a fixed frequency due to the imaginary part of the eigenvalues, while the behavior of node does not manifest a fixed frequency.

As a parameter is varied, the resting state can be changed to firing via a supercritical Hopf bifurcation, a subcritical Hopf (SubH) bifurcation, a SN bifurcation, or a SNIC bifurcation. The resting state corresponds to a stable focus for the Hopf bifurcation and a stable node for the SN/SNIC bifurcation. The SN/SINC bifurcation point exhibits a zero eigenvalue and the Hopf bifurcation manifests a pair of pure imaginary eigenvalues (zero real part). In addition to the Hopf and SN/SNIC bifurcation points, there are bifurcations mainly related to the limit cycle, such as the saddle homoclinic orbit (Hom) wherein a limit cycle contacted with a saddle. Except the above mentioned codim-1 bifurcations, codim-2 bifurcation such as the Bogdanov-Takens (BT) bifurcation with double zero eigenvalues, which is a transition point between the SN/SNIC bifurcation and the Hopf bifurcation, and the saddle-node homoclinic orbit bifurcation (SNHO), which is a switch point between the Hom and SNIC bifurcations, are also considered in the present paper.

Bifurcation diagrams are obtained using the dynamical software Matcont (Dhooge et al., 2003) and XPPAUT (Ermentrout, 2002).

## Results

### Simulations of the experimental observations related to *I*_*h*_

#### Post-inhibitory rebound (spike) induced by *I*_*h*_

**Figure 2:**
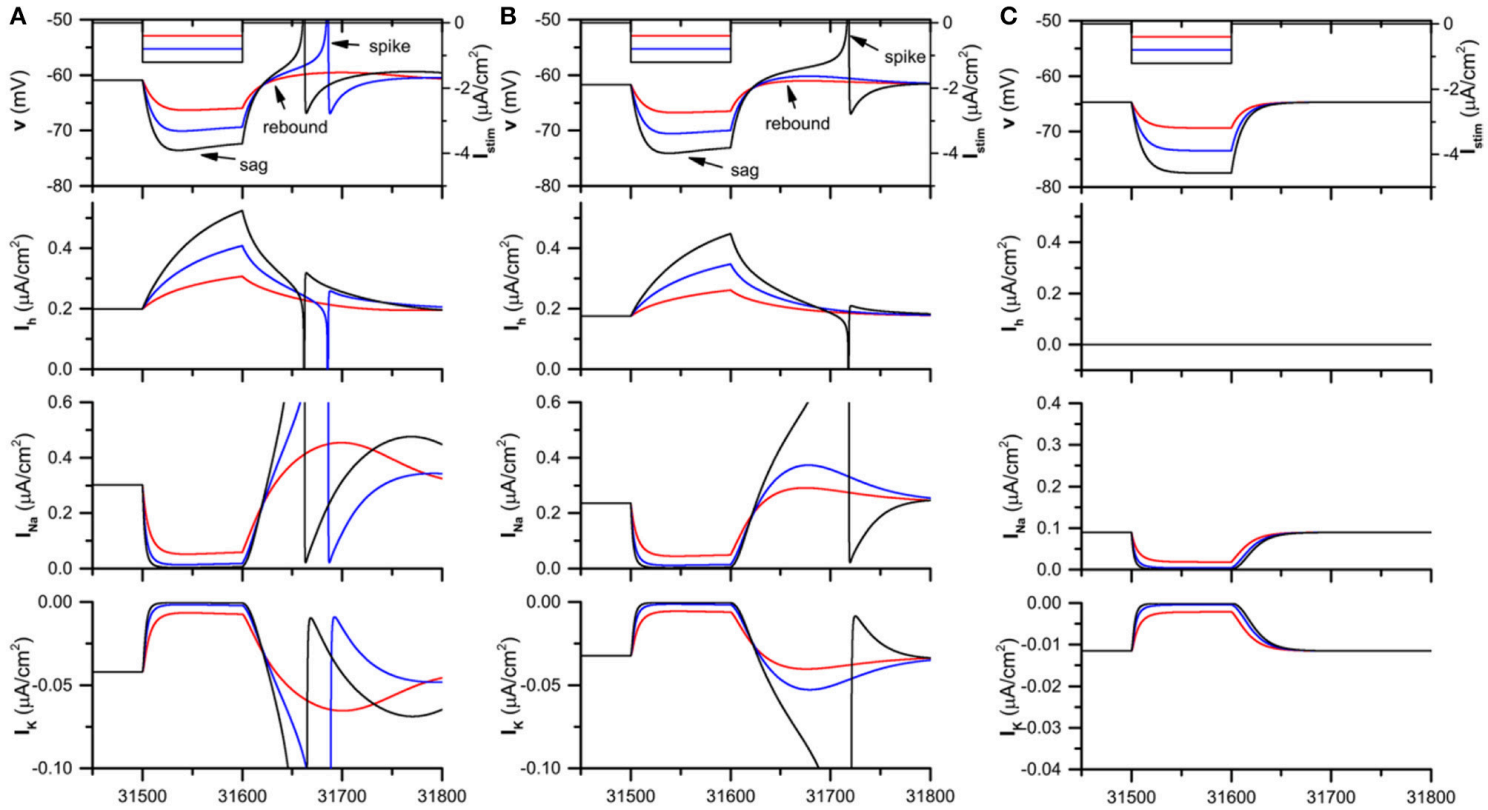
The *I*_*h*_ induced voltage sag and rebound (spike) and the evolution of the different currents in the theoretical neuron model stimulated by the hyperpolarization square current when $I_{app}=-0.05\mu \text{A}/\text{c}{\text{m}}^{2}$. **(A)** $g_{h}=0.05\text{mS}/\text{c}{\text{m}}^{2}$; **(B)** $g_{h}=0.04\text{mS}/\text{c}{\text{m}}^{2}$; **(C)** $g_{h}=0.0\text{mS}/\text{c}{\text{m}}^{2}$. The panels from top to bottom represent the hyperpolarization square current and the response of membrane potential, *I*_*h*_current, Na^+^ current, and K^+^ current. The amplitude of the hyperpolarization square current is −0.4 μA/cm^2^ (black), −0.8 μA/cm^2^ (blue), and −1.2 μA/cm^2^ (red).

When *I*_*app*_ = −0.05 μ*A*/*c*m^2^, the theoretical neuron model exhibits resting state when *g*_*h*_ = 0.05mS/cm^2^, 0.04 mS/cm^2^ (in the presence of *I*_*h*_), and 0.0 mS/cm^2^ (blockage of *I*_*h*_). When a hyperpolarization square current with duration 100 ms is injected to the theoretical model, the membrane potential exhibits different characteristics for different *g*_*h*_ levels, as shown in Figure 2.

For $g_{h}=0.05\text{ mS}/\text{c}{\text{m}}^{2}$, when the strength of the hyperpolarization square current is small (−0.4μA/cm^2^, red), both voltage sag, which appears at the initial phase of the hyperpolarization current, and post-inhibitory rebound phenomenon, which appears after the termination of the hyperpolarization current, are induced, as shown in the 1st panel of Figure 2A. When the strength increases to a middle level (−0.8μA/cm^2^, blue), the sag and rebound become strong and the rebound spike appears. When the strength increases to a high level (−1.2μA/cm^2^, black), both the voltage sag and rebound spike become strong, and the rebound spike appears earlier than that of the middle strength (−0.8μA/cm^2^).

For $g_{h}=0.04\text{ mS}/\text{c}{\text{m}}^{2}$, both voltage sag and rebound (spike) appear, and the rebound (spike) phenomenon becomes strong with increasing the current strength, which is similar to those of $g_{h}=0.05\text{ mS}/\text{c}{\text{m}}^{2}$. However, compared with $g_{h}=0.05\text{ mS}/\text{c}{\text{m}}^{2}$, both voltage sag and rebound (spike) become weak for $g_{h}=0.04\text{ mS}/\text{c}{\text{m}}^{2}$, as shown in the 1st panel of Figure 2B), which shows that the voltage sag and rebound (spike) become weak with decreasing *g*_*h*_. For example, when the current strength is middle (−0.8μA/cm^2^, blue), rebound spike appears for $g_{h}=0.05\text{ mS}/\text{c}{\text{m}}^{2}$ while not spike but rebound appears for $g_{h}=0.04\text{ mS}/\text{c}{\text{m}}^{2}$.

For $g_{h}=0.0\text{ mS}/\text{c}{\text{m}}^{2}$, which corresponds to the blockage of *I*_*h*_, both voltage sag and post-inhibitory rebound spike cannot be induced by the hyperpolarization square currents with the same strengths as Figures 2A,B, as depicted in the 1st panel of Figure 2C.

The results are consistent with the previous experimental investigations of *I*_*h*_, which were performed on the hippocampal pyramidal neurons and stratum oriens interneurons (Manseau et al., 2008; Ascoli et al., 2010; Zemankovics et al., 2010; Gastrein et al., 2011; Borel et al., 2013).

#### Enhancement of the resting potential and of the firing frequency induced by *I*_*h*_

**Figure 3:**
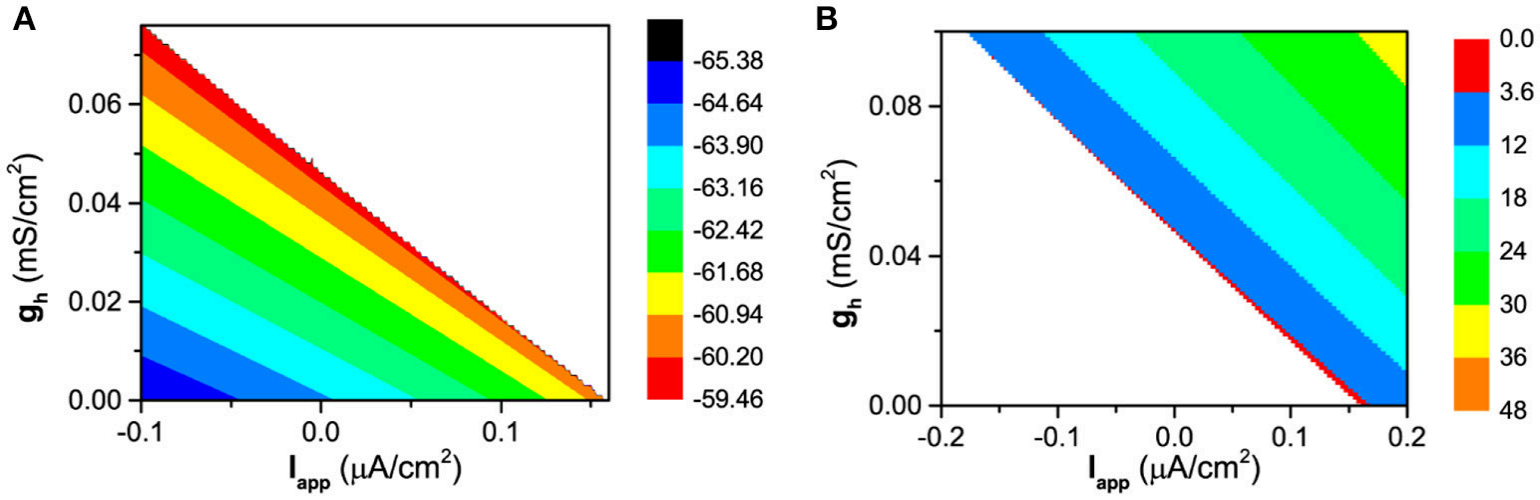
The changes of the dynamical behaviors of the theoretical model in (*I*_*app*_, *g*_*h*_) plane. **(A)** Membrane potential for the resting state; **(B)** Firing frequency for the firing behavior.

The changes of the membrane potential for the resting state and of the firing frequency for the firing behavior in (*I*_*app*_, *g*_*h*_) plane are shown in Figures 3A,B, respectively. The resting state locates at down-left half and the firing at up-right half of (*I*_*app*_, *g*_*h*_) plane. When *I*_*app*_ is fixed, with increasing *g*_*h*_, the membrane potential of the resting state increases, as shown in Figure 3A. The blank area corresponds to the firing behavior. When *I*_*app*_ is fixed, with increasing *g*_*h*_, the firing frequency increases, which means that the ISIs of firing decreases, as shown in Figure 3B. The blank area corresponds to the resting state. Both results are consistent with the biological experiments (Elgueta et al., 2015). In addition, the firing threshold for *I*_*app*_ decreases with increasing *g*_*h*_, which shows that *g*_*h*_ induces the decrease of the firing threshold for *I*_*app*_. Both enhancement of *I*_*h*_ and increase of *I*_*app*_ play the same positive roles in evoking firing, therefore, the increase of *g*_*h*_ leads to the decrease of firing threshold for *I*_*app*_.

#### *I*_*h*_ enhances the spike-timing precision

When *I*_*app*_ = 0.17 μA/cm^2^, the firing for both $g_{h}=0\text{mS}/\text{c}{\text{m}}^{2}$ (blockage of *I*_*h*_) and $g_{h}=0.02\text{mS}/\text{c}{\text{m}}^{2}$ (in presence of *I*_*h*_) in the deterministic model is period-1 and the *CV* of ISIs is 0.

**Figure 4:**
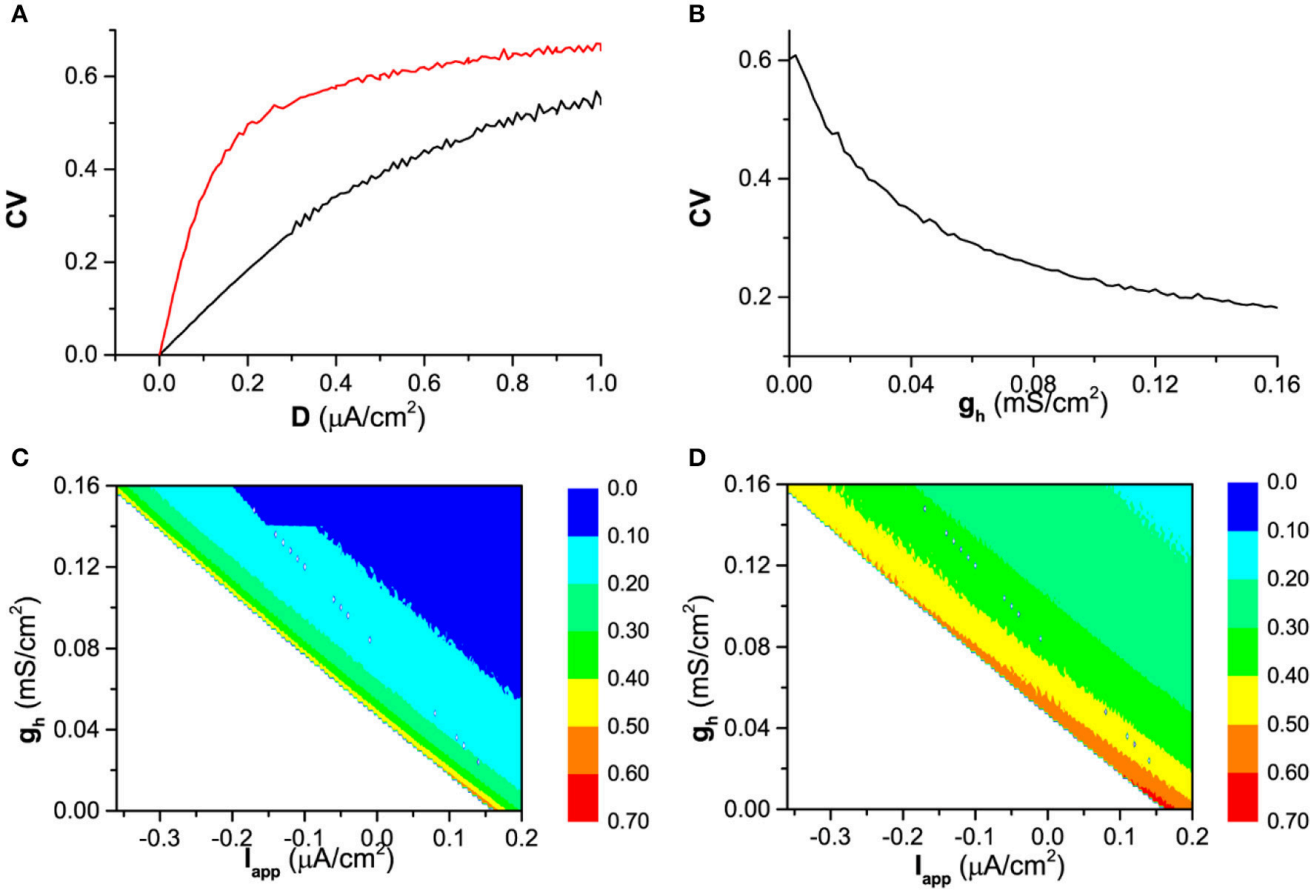
The spike-timing precision characterized by *CV* of ISIs. **(A)** The effect of noise intensity (*D*) on *CV* the in the presence of *I*_*h*_ (*g*_*h*_ = 0.02 mS/ cm^2^, black) and the absence of *I*_*h*_ ($g_{h}=0\text{mS}/\text{c}{\text{m}}^{2}$, red) when *I*_*app*_ = 0.17 μA/cm^2^; **(B)** The influence of *g*_*h*_ on *CV* when noise intensity is fixed (*D* = 0. 6 μA/cm^2^) when *I*_*app*_ = 0.17 μA/cm^2^; **(C)** *CV* in (*I*_*app*_, *g*_*h*_) plane when *D* = 0.2μA/cm^2^; **(D)** *CV* in (*I*_*app*_, *g*_*h*_) when *D* = 0.6μA/cm^2^.

When noise is introduced, the ISIs of the firing exhibit variability, which leads to a nonzero *CV*, for example, as shown in Figure 4A. With increasing noise intensity *D*, although *CV* for both $g_{h}=0\text{mS}/\text{c}{\text{m}}^{2}$ (red) and $g_{h}=0.02\text{mS}/\text{c}{\text{m}}^{2}$ (black) increases, *CV* for $g_{h}=0.02\text{mS}/\text{c}{\text{m}}^{2}$ is always higher than that for $g_{h}=0\text{mS}/\text{c}{\text{m}}^{2}$ in a large range of noise intensity (0 < *D* < 1 μA/cm^2^), which shows that spike-timing precision in the presence of *I*_*h*_ is higher than that of the blockage of *I*_*h*_.

The more spike-timing precision induced by *I*_*h*_ can also be found from Figure 4B. When noise intensity *D* is fixed, for example *D* = 0. 6 μA/cm^2^,*CV* decreases with increasing *g*_*h*_. The dependence of *CV* on both *I*_*app*_ and *g*_*h*_ are shown by the color scale in (*I*_*app*_, *g*_*h*_) plane in Figure 4C (*D* = 0.2μA/cm^2^) and Figure 4D (*D* = 0.6μA/cm^2^). For both noise intensities, *CV* decrease with increasing *g*_*h*_, which shows that *I*_*h*_ includes more spike-timing precision. No firing locates in down-left half of (*I*_*app*_, *g*_*h*_) plane (the blank area) and no *CV* is calculated.

**Figure 5:**
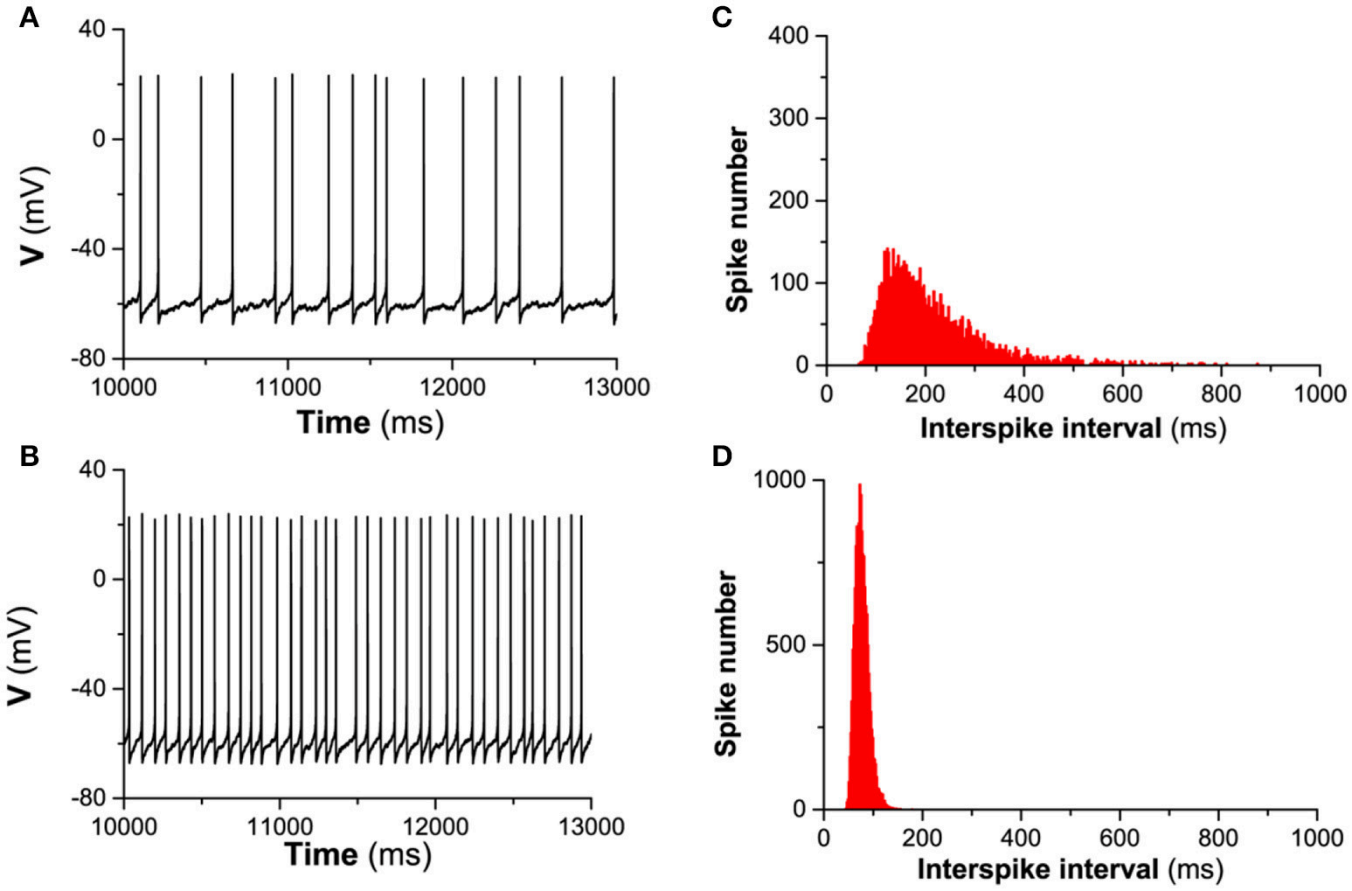
The influence of *I*_*h*_ on the spike-timing precision of the theoretical neuron model with noise (*D* = 0.2μA/cm^2^) when $I_{app}=0.17\mu \text{A}/\text{c}{\text{m}}^{2}$. **(A,B)**, spike trains corresponding to $g_{h}=0\text{mS}/\text{c}{\text{m}}^{2}$ and $g_{h}=0.02\text{mS}/\text{c}{\text{m}}^{2}$, respectively. **(C,D)**, ISIH corresponding to **(A,B)**, respectively.

The detailed difference of the spike-timing precision between the presence of *I*_*h*_ and the absence of *I*_*h*_ can be found from the spike trains of the firing and the ISI histogram (ISIH). For example, when *I*_*app*_ = 0.17 μA/cm^2^ and *D* = 0.2μA/cm^2^, the spike trains for $g_{h}=0\text{mS}/\text{c}{\text{m}}^{2}$ and $g_{h}=0.02\text{mS}/\text{c}{\text{m}}^{2}$ are shown in Figures 5A,B), respectively, and the ISIH for $g_{h}=0\text{mS}/\text{c}{\text{m}}^{2}$ and $g_{h}=0.02\text{mS}/\text{c}{\text{m}}^{2}$ is illustrated in Figures 5C,D), respectively, which closely matches the experimental observations on the hippocampal pyramidal neurons and stratum oriens interneurons (Gastrein et al., 2011). The mean value of ISIs is 208.99 ms for $g_{h}=0\text{mS}/\text{c}{\text{m}}^{2}$ and 76.98 ms for $g_{h}=0.02\text{mS}/\text{c}{\text{m}}^{2}$, respectively. The standard deviation (*STD*) of ISIs is 102.24 ms for $g_{h}=0\text{mS}/\text{c}{\text{m}}^{2}$ and 14.09 ms for $g_{h}=0.02\text{mS}/\text{c}{\text{m}}^{2}$, respectively. The *CV* of the ISIs is 0.494 for $g_{h}=0\text{mS}/\text{c}{\text{m}}^{2}$ and 0.183 for $g_{h}=0.02\text{mS}/\text{c}{\text{m}}^{2}$, respectively. The result indicates that *I*_*h*_ reduces the variability of ISIs, i.e., enhances the precision of the spike-timing, which can also be found from the ISIH depicted in Figures 5C,D). Compared with Figure 5D (blockage of *I*_*h*_), ISIH becomes broader and lower for Figure 5C (in the presence of *I*_*h*_), which also shows that *I*_*h*_ can enhance the spike-timing precision.

#### Subthreshold resonance induced by *I*_*h*_

Two cases of the subthreshold resonance for different *I*_*app*_ values are simulated.

**Figure 6:**
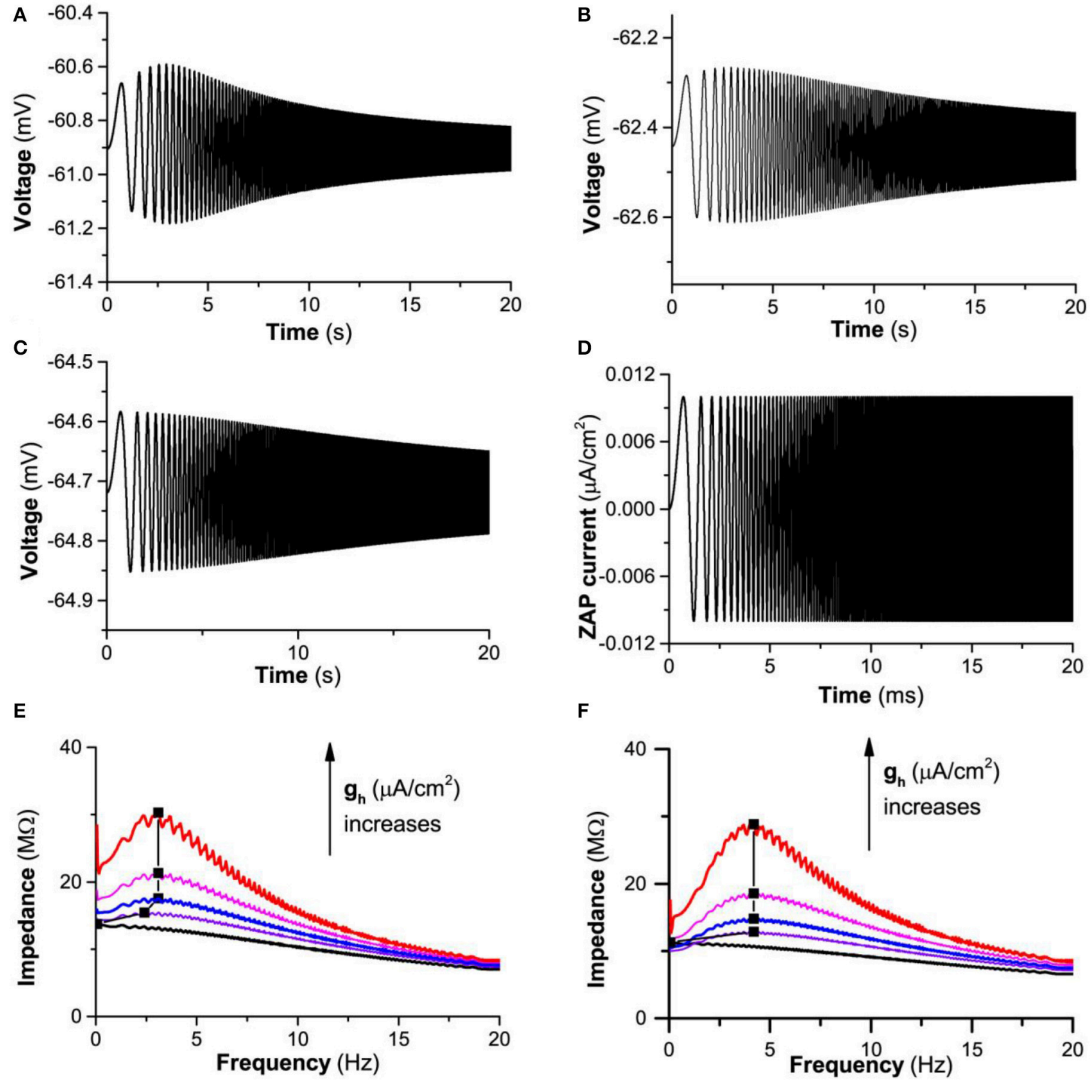
The influence of *I*_*h*_ on the subthreshold resonance of the theoretical neuron model stimulated by a subthreshold “ZAP” current. $I_{app}=-0.05\mu \text{A}/\text{c}{\text{m}}^{2}$. Voltage response: **(A)** *g*_*h*_ = 0.05 mS/cm^2^; **(B)** *g*_*h*_ = 0.03 mS/cm^2^; **(C)** *g*_*h*_ = 0.0 mS/cm^2^; **(D)** “ZAP” current; **(E)** Impedance vs. frequency at different *g*_*h*_ levels; From top to bottom, *g*_*h*_ = 0.05, 0.04, 0.03, 0.02, and 0.0 mS/cm^2^. **(F)** Impedance vs. frequency at different *g*_*h*_ levels for $I_{app}=-0.3\mu \text{A}/\text{c}{\text{m}}^{2}$; From top to bottom, *g*_*h*_ = 0.12, 0.1, 0.08, 0.06, and 0.0 mS/cm^2^.

When $I_{app}=-0.05\mu \text{A}/\text{c}{\text{m}}^{2}$, the theoretical model exhibits resting state when *g*_*h*_ = 0.0 mS/cm^2^, 0.03 mS/cm^2^, and 0.05 mS/cm^2^. When a subthreshold “ZAP” current (Figure 6D) is applied, the membrane potential exhibits different responses at different *g*_*h*_ values, as shown in Figure 6A (*g*_*h*_ = 0.05 mS/cm^2^), Figure 6B (*g*_*h*_ = 0.03 mS/cm^2^), and Figure 6C (*g*_*h*_ = 0.0 mS/cm^2^). When *g*_*h*_ = 0.05 mS/cm^2^, the amplitude of the oscillation increases firstly and then decreases with respect to the increase of time/frequency, correspondingly, the impedance profile exhibits an inverse “U” shape, as shown by the first line from the top (red) in Figure 6E, showing that a “typical”-resonance appears. The resonance frequency is around 3.1 Hz. When *g*_*h*_ decreases to 0.03 mS/cm^2^, the amplitude of the oscillation exhibits a slight increase at first and then a decrease with increasing time/frequency, and the height of the inverse “U” shape of the impedance profile becomes short, which shows that the resonance becomes weak, as shown by the third line (blue) from the top in Figure 6E. In the present paper, such a resonance is called as “weak”-resonance. When *g*_*h*_ decreases to 0.0 mS/cm^2^, the amplitude of the oscillation decreases with increasing time/frequency and the phenomenon of resonance disappears, called as non-resonance, as shown by the last line (black) from the top in Figure 6E. With decreasing *g*_*h*_ from 0.05 mS/cm^2^, to 0.04mS/cm^2^, to 0.03 mS/cm^2^, to 0.02 mS/cm^2^, and to 0.0 mS/cm^2^, the maximal impedance decreases, as shown by the black square in Figure 6E. The result is consistent with the experimental observations in many types of neurons such as the stratum oriens interneuron (Zemankovics et al., 2010; Gastrein et al., 2011; Pavlov et al., 2011; Borel et al., 2013; Gonzalez et al., 2015).

Another representative similar to *I*_*app*_ = −0.05μA/cm^2^ is shown in Figure 6F (*I*_*app*_ = −0.3 μA/cm^2^). The upper 2 lines (*g*_*h*_ = 0.12 and 0.1 mS/cm^2^), middle 2 lines (*g*_*h*_ = 0.08 and 0.06 mS/cm^2^), and lowest line (*g*_*h*_ = 0.0 mS/cm^2^) for impedance vs. frequency exhibit “typical”-, “weak”-, and non-resonances.

**Figure 7:**
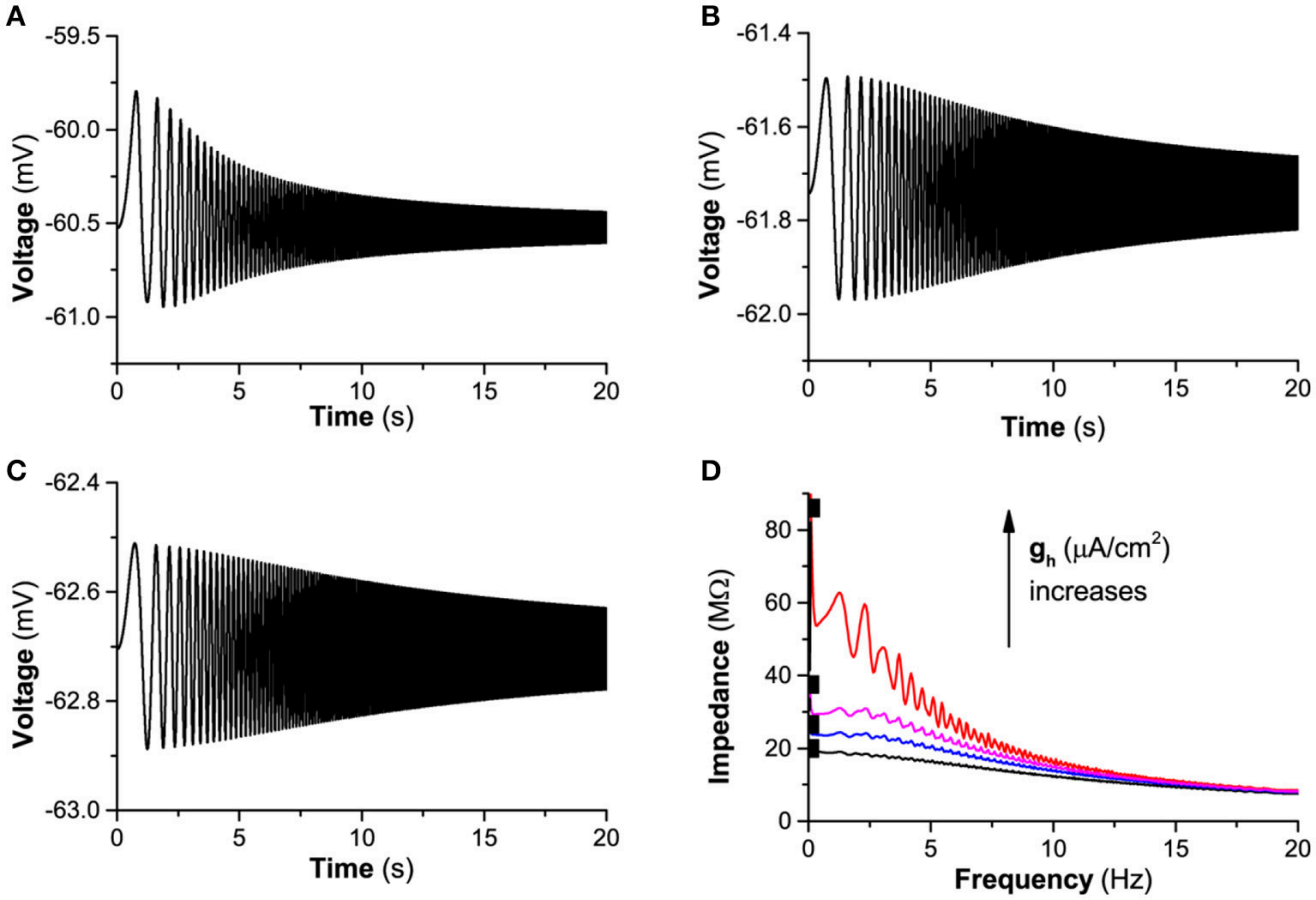
The influence of *I*_*h*_ on the subthreshold resonance of the theoretical neuron model stimulated by a subthreshold “ZAP” current when $I_{app}=0.08\mu \text{A}/\text{c}{\text{m}}^{2}$. Voltage response: **(A)** *g*_*h*_ = 0.02 mS/cm^2^; **(B)** *g*_*h*_ = 0.01 mS/cm^2^; **(C)** *g*_*h*_ = 0.0 mS/cm^2^; **(D)** Impedance vs. frequency at different *g*_*h*_ levels; From top to bottom, *g*_*h*_ = 0.02 (red), 0.15 (pink), *g*_*h*_ = 0.01 (blue), and *g*_*h*_ = 0.0 mS/cm^2^ (black).

When $I_{app}=0.08\mu \text{A}/\text{c}{\text{m}}^{2}$, the response of the membrane potential to “ZAP” current and resonance exhibits characteristic different from $I_{app}=-0.05\mu \text{A}/\text{c}{\text{m}}^{2}$ and *I*_*app*_ = −0.3 μA/cm^2^, as shown in Figure 7. From *g*_*h*_ = 0.02 mS/cm^2^ to 0.01 mS/cm^2^, and to 0.0 mS/cm^2^, the responses of the membrane potential are shown in Figures 7A–C). The amplitude of the oscillation of the membrane potential decreases with increasing time/frequency, which shows that not “typical”-resonance but non- or “weak”-resonance appears, as shown by the red (*g*_*h*_ = 0.02 mS/cm^2^), blue (*g*_*h*_ = 0.01 mS/cm^2^), and black lines (*g*_*h*_ = 0.0 mS/cm^2^) in Figure 7D. With decreasing *g*_*h*_, the maximal impedance decreases, as shown the black square in Figure 7D.

### Bifurcation analysis

#### One-parameter bifurcation

**Figure 8:**
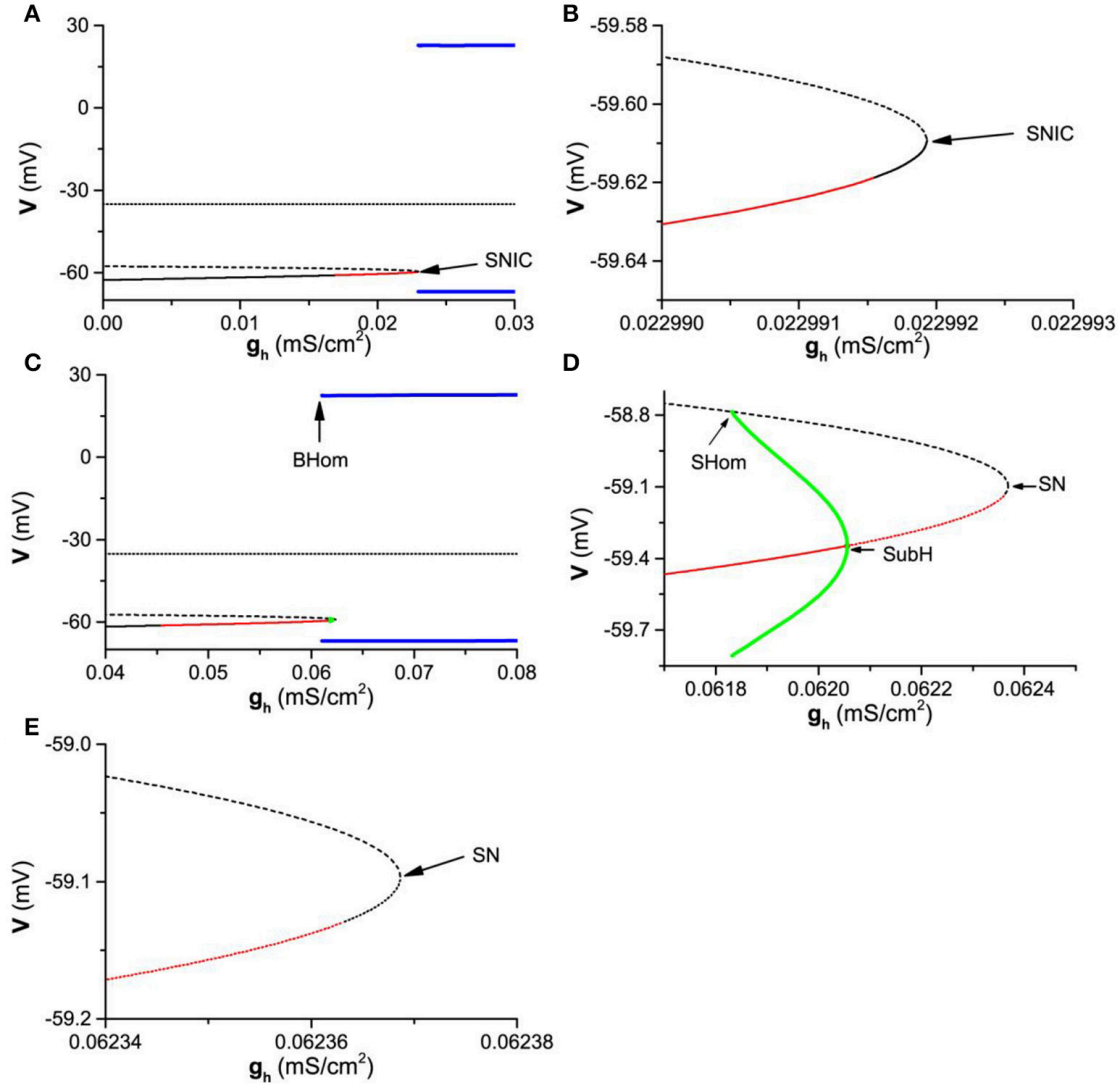
One-parameter bifurcations of the membrane potentials (*V*) with respect to *g*_*h*_ at different *I*_*app*_ levels. **(A)** $I_{app}=0.08\mu \text{A}/\text{c}{\text{m}}^{2}$; **(B)** Partial enlargement of the middle and lower branches of the equilibrium of **(A)** near SNIC; **(C)** $I_{app}=-0.05\mu \text{A}/\text{c}{\text{m}}^{2}$; **(D)** Partial enlargement of **(C)** around SN and SubH; **(E)** Partial enlargement of **(D)**. The black solid, dashed, and dotted curves represent the stable node, unstable node, and saddle, respectively. The red solid and dashed curves represent the stable and unstable focus, respectively. The upper (lower) blue line represents the maximal (minimal) value of the stable limit cycle corresponding to firing. The upper (lower) green curve is the maximal (minimal) value of the membrane potential of the unstable limit cycle. SNIC and SN represent saddle-node bifurcation on invariant circle and saddle-node bifurcation, respectively. SHom and BHom are the small and big saddle homoclinic orbits, respectively. SubH represents the subcritical Hopf bifurcation.

Considering that the subthreshold resonance for $I_{app}=0.08\mu \text{A}/\text{c}{\text{m}}^{2}$ exhibits characteristics different from that of $I_{app}=-0.05\mu \text{A}/\text{c}{\text{m}}^{2}$, the bifurcations with respect to *g*_*h*_ for $I_{app}=0.08\mu \text{A}/\text{c}{\text{m}}^{2}$ and *I*_*app*_ = −0.05 are calculated and manifest different dynamics, as shown in Figure 8.

For $I_{app}=0.08\mu \text{A}/\text{c}{\text{m}}^{2}$, the bifurcation of the theoretical model with respect to *g*_*h*_ are shown in Figures 8A,B (Figures 8B is the partial enlargement of Figures 8A). The theoretical neuron model exhibits firing/stable limit cycle (blue bold lines) when $g_{h}\geq 0.0229919\text{mS}/\text{c}{\text{m}}^{2}$. The upper and lower blue lines represent the maximal and minimal values of the membrane potential of firing. The equilibrium contains three branches (dotted, dashed, or thin solid lines). The upper branch (dotted line) and middle branch (dashed line) are unstable focus and saddle (unstable), respectively, and are not associated with the resting state. The lower branch (thin solid line) is related the resting state and composed of three parts, a stable node ($g_{h}<0.0169329\text{mS}/\text{c}{\text{m}}^{2}$, black solid line left to the red solid line), a stable focus ($0.0169329<g_{h}<0.0229915\text{mS}/\text{c}{\text{m}}^{2}$, red solid line), and a stable node ($0.0229915<g_{h}<0.0229919\text{mS}/\text{c}{\text{m}}^{2}$, black solid line right to the red solid line). Therefore, the resting state contains two dynamical behaviors, stable node and stable focus. There is a SNIC bifurcation at $g_{h}\approx 0.0229919\text{mS}/\text{c}{\text{m}}^{2}$, wherein a stable node (right part of the lower branch, black solid line right to the red line) contacts with a saddle (middle branch, dotted line) to disappear and a limit cycle (blue) appears. As *g*_*h*_ changes across the SNIC bifurcation point, the transition between the resting state corresponding to a stable node and the firing corresponding to a stable limit cycle happens.

Therefore, with increasing *g*_*h*_, there are four stable behaviors and 3 bifurcation/transition points. The four behaviors are stable node, stable focus, stable node, and firing (stable limit cycle), and the 3 bifurcation/transition points are the transition point from the stable node to stable focus, transition point from the stable focus to stable node, and the SNIC bifurcation point.

For $I_{app}=-0.05\mu \text{A}/\text{c}{\text{m}}^{2}$, the bifurcations are different from those of $I_{app}=0.08\mu \text{A}/\text{c}{\text{m}}^{2}$, as shown in Figures 8C–E) (Figure 8D is the partial enlargement of Figure 8C, and Figure 8E is the partial enlargement of Figure 8D). Except for the firing/stable limit cycle (blue bold lines) and 3 branches of the equilibrium points (dashed or thin solid lines), a small unstable limit cycle (green) appears. The lower branch of the equilibrium point associated with the resting state is different from that of $I_{app}=0.08\mu \text{A}/\text{c}{\text{m}}^{2}$. With increasing *g*_*h*_, the equilibrium (lower branch) is stable node ($g_{h}<0.0454454\text{mS}/\text{c}{\text{m}}^{2}$, black solid line), stable focus ($0.0454454<g_{h}<0.0620557\text{mS}/\text{c}{\text{m}}^{2}$, red solid line), unstable focus ($0.0620557<g_{h}<0.0623584\text{mS}/\text{c}{\text{m}}^{2}$, red dashed line), and unstable node ($0.0623584<g_{h}<0.0623686\text{mS}/\text{c}{\text{m}}^{2}$, black dashed line). Furthermore, there are more types of bifurcations for $I_{app}=-0.05\mu \text{A}/\text{c}{\text{m}}^{2}$. For example, the firing/stable limit cycle related to a big saddle homoclinic orbit (BHom) appears at $g_{h}\approx 0.0610595\text{mS}/\text{c}{\text{m}}^{2}$, wherein a stable limit cycle with large amplitude (blue) contacts with a saddle (middle branch of the equilibrium). Other three types of bifurcations are subcritical Hopf (SubH) bifurcation at $g_{h}\approx 0.0620557\text{mS}/\text{c}{\text{m}}^{2}$, saddle-node (SN) bifurcation at $g_{h}\approx 0.0623686\text{mS}/\text{c}{\text{m}}^{2}$, and small saddle homoclinic orbit (SHom) at *g*_*h*_ ≈ 0.061832mS/cm^2^. The stable focus (red solid line) changes to unstable focus (red dashed line) via the SubH bifurcation and an unstable limit cycle with small amplitude (green) appears left to the SubH. The unstable limit cycle with small amplitude (green) contacts with the middle branch of the equilibrium (saddle) to form a SHom and disappears. The unstable node at the lower branch of the equilibrium contacts with the saddle (middle branch) to form a SN bifurcation and both equilibriums disappear.

As *g*_*h*_ increases across the SubH bifurcation point ($g_{h}\approx 0.0620557\text{mS}/\text{c}{\text{m}}^{2}$), the resting state corresponding to a focus (red solid line) changes to the firing corresponding to a stable limit cycle (blue line). As *g*_*h*_ decreases across the BHom bifurcation point ($g_{h}\approx 0.0610595\text{mS}/\text{c}{\text{m}}^{2}$), the firing corresponding to a stable limit cycle (blue line) changes to the resting state corresponding to a focus (red solid line). Therefore, bistability or coexistence of the resting state and firing lies between the BHom and SubH bifurcation points, which closely matches the experimental observations in the Purkinje cells (Engbers et al., 2013).

With increasing *g*_*h*_, there are 6 bifurcation/transition points, the transition point from the stable node to stable focus, the BHom point, the SHom point, the SubH point, the transition point from the unstable focus to unstable node, and the SN bifurcation point related to the unstable node. There are 4 stable behaviors, the stable node, the stable focus, the coexistence of the resting state and firing, and the firing. The 3 bifurcation/transition points related to the stable dynamical behaviors, the transition point from the stable node to focus, the BHom point, and the SubH point, form the border of the parameter region between the four stable dynamical behaviors.

#### Two-parameter bifurcation

**Figure 9:**
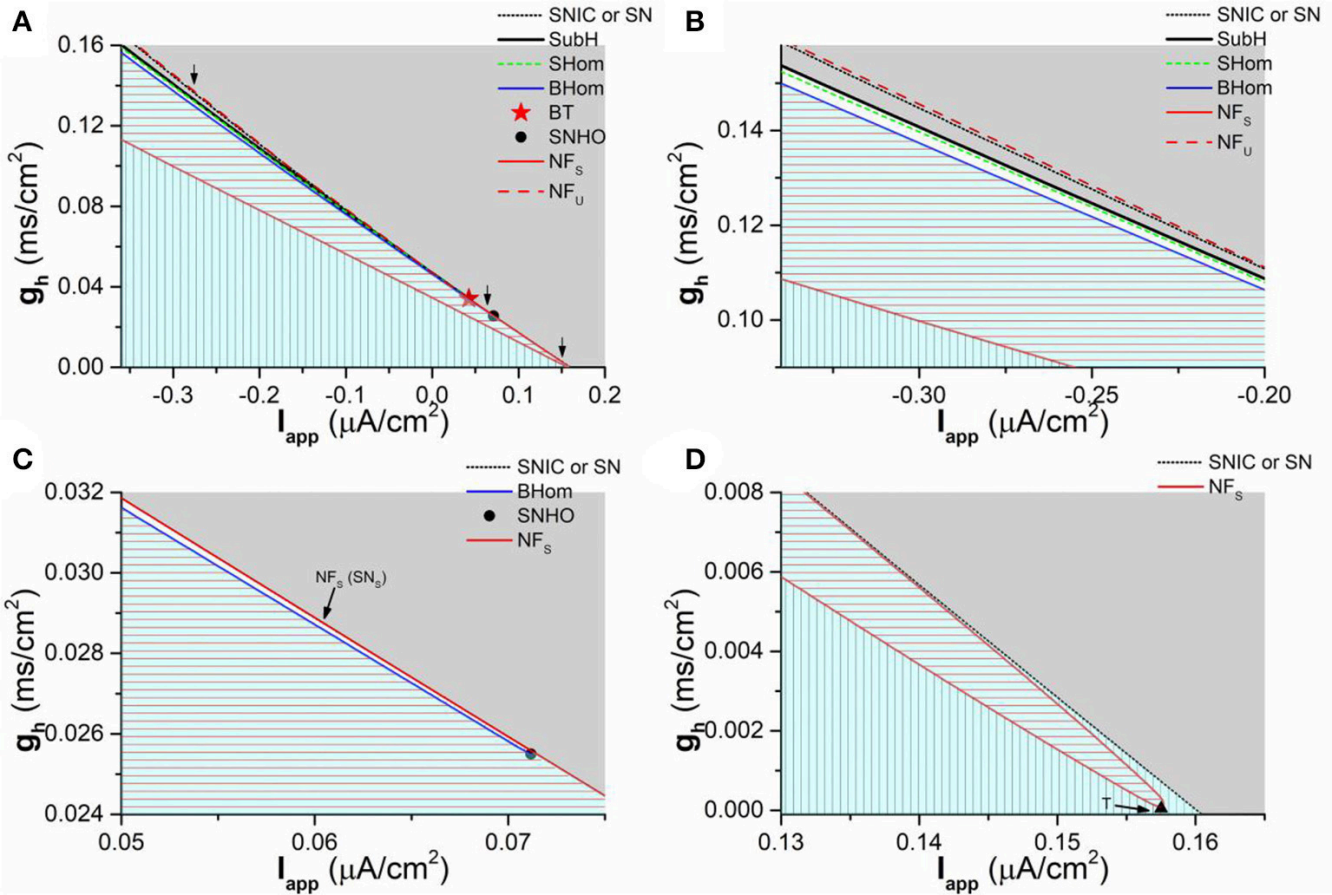
Two-parameter bifurcations in (*g*_*h*_, *I*_*app*_) plane. **(A)** Global view of the bifurcation diagram; **(B)** Partial enlargement diagram of a part of **(A)** around the first arrow (left to BT point); **(C)** Partial enlargement diagram of a part of **(A)** around the second arrow (between the BT point and SNHO point); **(D)** Partial enlargement diagram of a part of **(A)** around the last arrow (right to the SNHO point). SNIC and SN represent saddle-node bifurcation on invariant circle and saddle-node bifurcation, respectively. SHom and BHom are small and big saddle homoclinic orbits, respectively. SubH is the subcritical Hopf bifurcation. BT is Bogdanov-Takens bifurcation. SNHO is saddle-node homoclinic orbit bifurcation. SN_S_ is a part of SN curve related to the stable node. NF_U_ and NF_S_ represent the NF curve related to the stable focus/node and unstable focus/node, respectively. The stable behaviors within 4 sub-regions are firing (Gray), coexistence of firing and resting state (White area), stable focus (red horizontal lines), and stable node (vertical lines), respectively.

To investigate the comprehensive roles of *I*_*h*_ on the dynamical behaviors, bifurcations, and transitions between different kinds of bifurcations, the bifurcation diagram in (*I*_*app*_, *g*_*h*_) plane is acquired, as shown in Figure 9. In (*I*_*app*_, *g*_*h*_) plane, the codimension-1 bifurcation or transition points connect to form codimension-1 bifurcation or transition curves, which is the border of the parameter region between different dynamical behaviors. There are multiple codimension-1 bifurcation or transition curves, such as the SubH curve, BHom curve, SN curve, SNIC curve, and transition curve between node and focus which is labeled as NF curve. NF_U_ (dashed red line) and NF_S_ (solid red line) curves represent the part of the NF curve related to stable focus/node and unstable focus/node, respectively. SN_S_ curve is the part of the SN curve related to the stable node. The intersection point between different kinds of codimension-1 bifurcation curves is the codimension-2 bifurcation point, which is the key point to characterize the transition between different kinds of bifurcations. There are 2 codimmension-2 bifurcation points. One is Bogdanov-Takens (BT) point, which is labeled with a star and locates at $I_{app}\approx 0.0432\mu \text{A}/\text{c}{\text{m}}^{2}$ and $g_{h}\approx 0.03413\text{mS}/\text{c}{\text{m}}^{2}$. The other is the saddle-node homoclinic orbit (SNHO) bifurcation point, which is labeled with a black circle point and locates at $I_{app}\approx 0.07118\mu \text{A}/\text{c}{\text{m}}^{2}$ and $g_{h}\approx 0.02549\text{mS}/\text{c}{\text{m}}^{2}$. In addition, the NF_S_ curve (red line) is composed of the upper and lower branches and the connection point between two branches is called as T point (*I*_*app*_ ≈ 0.15751μA/cm^2^ and *g*_*h*_ ≈ 0.00001mS/cm^2^), which is labeled with a black triangle (Figure 9D).

The enlargement of 3 part of the bifurcations from resting state to firing, which are labeled with arrows in Figure 9A, are shown in Figure 9B (Left to BT point), Figure 9C (between BT and SNHO points), and Figure 9D (right to SNHO point). The (*I*_*app*_, *g*_*h*_) plane is divided into 4 parts by the codimension-1 bifurcation or transition curves. From top to bottom, the stable behaviors within the 4 sub-regions are firing (gray), coexistence of firing and resting state (blank area in Figures 9B,C, too small to be seen in Figure 9A), stable focus (red horizontal lines), and stable node (vertical lines), as shown in Figure 9. The border of the parameter region of the 4 stable behaviors composed of codimension-1 bifurcation or transition curve and codimension-2 bifurcation points is shown in Table 1. From left to right, the bottom border of the firing is SubH curve (black line, Figures 9A,B), BT point, SN curve (black dashed line in Figures 9A–C, very close to the upper branch of the NF_S_ curve in Figure 9C), SNHO point, SNIC curve (black dashed line in Figures 9A–D). This border left to the SNHO point is the top border of the coexistence behavior, and the bottom border of the coexistence is the BHom curve (blue line in Figures 9A–C) ended at SNHO point. The top border of the stable focus region is composed of the BHom curve (blue line in Figures 9A–C) and the upper branch of the NF_S_ curve (red line in Figures 9C,D) between the BT point and T point, and the bottom border is the lower branch of the NF_S_ curve (red line in Figures 9A–D). The stable node locates in the remained area (Vertical line in Figures 9A,B,D).

**Table 1:** The top or bottom border of the parameter region of the four stable behaviors.

**Stable behaviors**	**Curve**	**BT point**	**Curve**	**SNHO point**	**Curve**	**T point**	**Area**
Firing	SubH	Pass	SN	Pass	SNIC		
Coexistence	SubH	Pass	SN	End			/
	BHom			End			
Focus	BHom			Pass	Upper NF_S_	End	/
	Lower NF_S_					End	/
Node	The remained area						

The upper branch of the transition curve between the stable node and focus (NF_S_ curve) intersects with the codimension-1 SN bifurcation curve, SubH curve, and SHom curve at the BT point, which shows that BT point is related to both Hopf and SN/SNIC bifurcations, i.e., the transition point between Hopf and SN/SNIC bifurcations. The SNHO point is the intersection point between the bifurcation curves of SN, BHom, and SNIC. Other bifurcation (SHom and NF_U_) curves not related to the stable behaviors, which are close to some of the bifurcation curves mentioned above, are not addressed in the present paper.

In summary, two obvious characteristics can be found from the bifurcation diagram. One is that the codimension-1 bifurcation curve or the border between the firing and resting state show a negative slope in the (*I*_*app*_, *g*_*h*_) plane, which correspond to the firing threshold shown in Figure 3. The firing behavior and the resting state locate up-right half and down-left half of (*I*_*app*_, *g*_*h*_) plane, respectively. The result shows that increasing *g*_*h*_ means far away from the bifurcation point for the firing behavior and approaching bifurcation point for the resting state corresponding to a stable focus or stable node. The other is that the distribution of the dynamical behaviors in the parameter region left and right to the BT point is different. Left to the BT point, the stable focus locates upper to the stable node. With increasing *I*_*app*_, the parameter region of the stable focus becomes narrow. Down-right to the BT point, the parameter region of the resting state and stable focus becomes very narrow and at last the stable focus disappears with increasing *I*_*app*_. The behavior upper, lower, and right to the stable focus is stable node (Figure 9D).

### Dynamical mechanism of *I*_*h*_ induced resonance and spike-timing precision, and rebound

#### Dynamical mechanism of *I*_*h*_ induced subthreshold resonance

Firstly, the parameter regions for the two cases of the subthreshold resonance can be identified. Two different cases of subthreshold resonance shown in subsection 3.1.4 locate at left (*I*_*app*_ = −0.05 μA/cm^2^ and *I*_*app*_ = −0.3 μA/cm^2^, Figure 6) and right (*I*_*app*_ = 0.08 μA/cm^2^, Figure 7) to the BT point, respectively, which implies that the BT point plays very important roles in the identification of dynamical mechanism of the subthreshold resonance.

Secondly, with help of the distribution of the stable focus and node left to the BT point, the “typical” -, “weak” -, and non-resonance can be well understood. The “typical”-resonance appears at large *g*_*h*_ value (*g*_*h*_ = 0.05 mS/cm^2^ for *I*_*app*_ = −0.05 μA/cm^2^, *g*_*h*_ = 0.1 mS/cm^2^ and 0.12 mS/cm^2^ for *I*_*app*_ = −0.3 μA/cm^2^), i.e., within the parameter region of the stable focus. The non-resonance appears at *g*_*h*_ = 0 mS/cm^2^ (*I*_*app*_ = −0.05 μA/cm^2^ and *I*_*app*_ = −0.3 μA/cm^2^), i.e., within the parameter region of the stable node. The “weak” -resonance appears near the transition curve between the stable focus and node (*g*_*h*_ = 0.02, 0.03 and 0.04 mS/cm^2^for *I*_*app*_ = −0.05 μA/cm^2^, *g*_*h*_ = 0.06 and 0.08 mS/cm^2^ for *I*_*app*_ = −0.3 μA/cm^2^). The results show that the “typical”-resonance in the presence of *I*_*h*_ is related to the stable focus, and the non-resonance in the absence of *I*_*h*_ (blockage of *I*_*h*_) associated with the stable node. Therefore, *I*_*h*_ induces a transition from the stable node to the stable focus. In nonlinear dynamics, Hopf bifurcation related to the stable focus/firing and SN/SNIC bifurcation associated with the stable node/firing. Therefore, the transition point between the Hopf bifurcation and SN/SNIC bifurcation, i.e., the BT point, exists in the neuronal system with *I*_*h*_.

**Figure 10:**
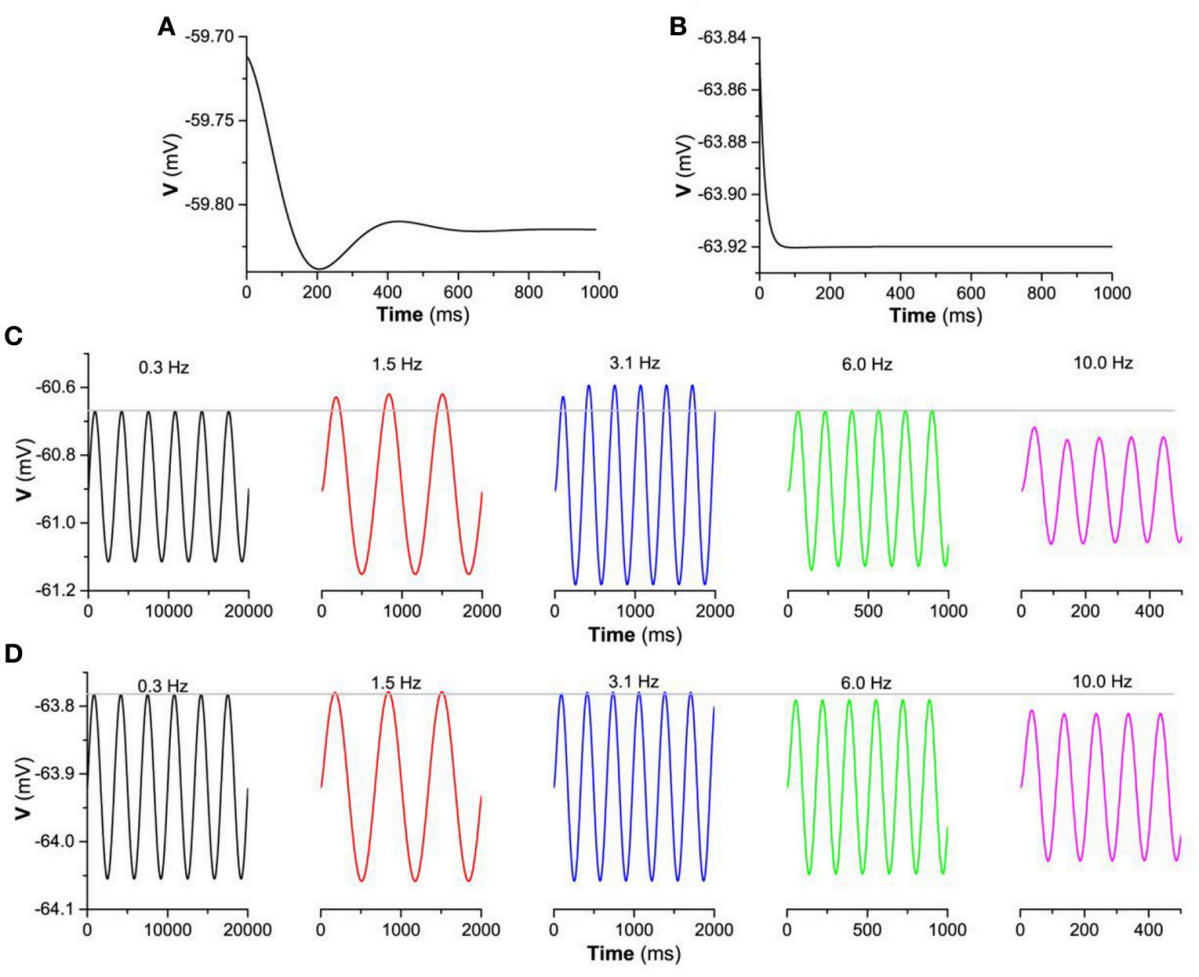
The membrane potential of the stable focus and stable node when perturbed and the responses to a periodic stimulus with single frequency for $I_{app}=-0.05\mu \text{A}/\text{c}{\text{m}}^{2}$. The membrane potential when perturbed. **(A)** Stable focus when *g*_*h*_ = 0.05 mS/cm^2^; **(B)** Stable node when; *g*_*h*_ = 0.01 mS/cm^2^; The responses of the membrane potential to periodic stimulus *g*_*h*_ = 0.01 mS/cm^2^ current a sin 2π *f*_1_*t* ($a=0.01\mu \text{A}/\text{c}{\text{m}}^{2}\;f_{1}$ is the frequency); Voltage response to periodic stimulus with singe frequency. **(C)** Stable focus when $g_{h}=0.05\text{mS}/\text{c}{\text{m}}^{2}$; **(D)** Stable focus when $g_{h}=0.01\text{mS}/\text{c}{\text{m}}^{2}$.

Thirdly, the “typical”-resonance for the stable focus or non-resonance for the stable node and the difference between them are origin from the distinction of the frequency response due to eigenvalue. The eigenvalues for the stable focus are complex numbers and for the stable node are real numbers. When disturbed, the behavior of a stable focus is a damped subthreshold oscillation with a fixed frequency which is related to the imaginary part of the complex eigenvalues, as shown in Figure 10A ($g_{h}=0.05\text{mS}/\text{c}{\text{m}}^{2},I_{app}=-0.05\mu \text{A}/\text{c}{\text{m}}^{2}$). However, the behavior of a stable node after perturbed exhibits no oscillations, as shown in Figure 10B ($g_{h}=0.01mS/cm^{2},I_{app}=-0.05\mu \text{A}/\text{c}{\text{m}}^{2}$). Such two different behaviors exhibit different frequency responses to the periodic stimulus with single frequency. For a stable focus, the oscillation amplitude of the membrane potential reaches maximal when the stimulus frequency is close to the fixed frequency of the focus. For example, when stimulus frequency is 3.1 Hz, the oscillation amplitude is higher than those of other frequencies, as shown in Figure 10C. For a stable node, the oscillation amplitude of the membrane potential remains nearly unchanged at low frequency and then decreases with increasing the stimulus frequency, as shown in Figure 10D. The periodic stimulus current is *a*sin2π*f*_1_*t* (*a* = 0.01μA/cm^2^, *f*_1_ is the frequency). The different frequency responses between the stable focus and stable node are the fundamental base of the “typical”-resonance and non-resonance phenomenon induced by the “ZAP” current.

**Figure 11:**
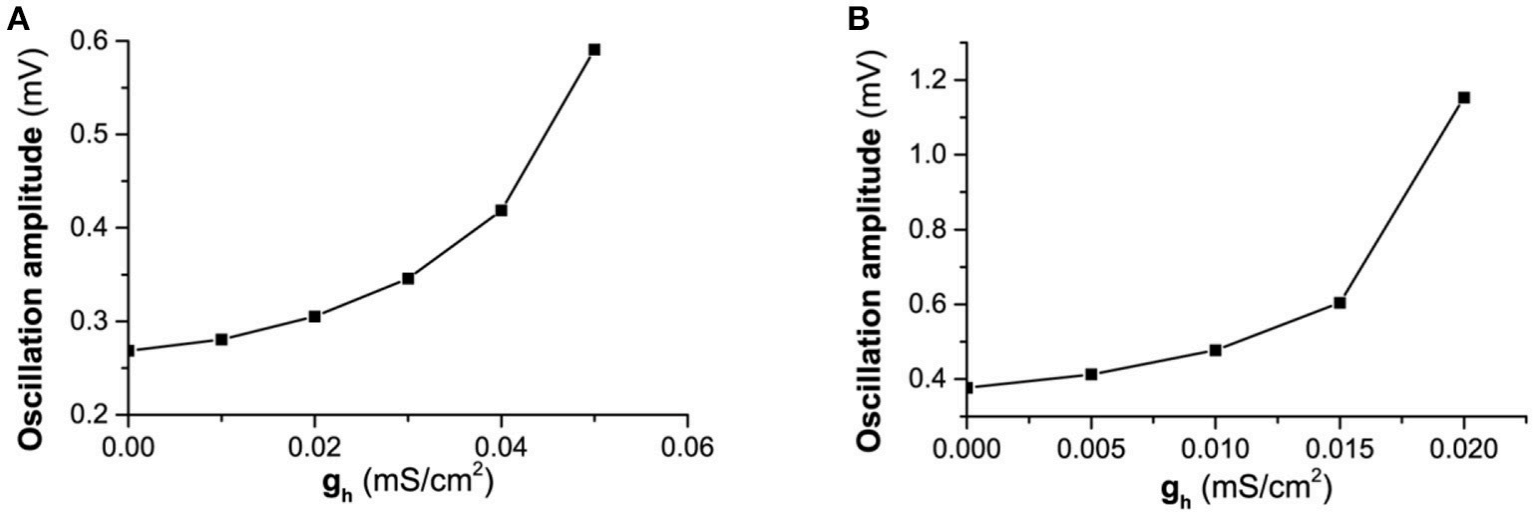
The changes of the difference between the maximal and minimal values of the membrane potential stimulated by a “ZAP” current with increasing *g*_*h*_. **(A)** $I_{app}=-0.05\mu \text{A}/\text{c}{\text{m}}^{2}$; **(B)** $I_{app}=0.08\mu \text{A}/\text{c}{\text{m}}^{2}$.

Fourthly, another important characteristic of the subthreshold resonance (Figures 6E,F,D) is that the maximal impedance increases with increasing *g*_*h*_ when *I*_*app*_ is fixed, which can be well understood with the bifurcation theory and the changes of the membrane potential shown in Figure 3A. With increasing *g*_*h*_, the membrane potential increases. When *I*_*app*_ is fixed, increasing *g*_*h*_ means that approaching the bifurcation point from resting state to firing, and the oscillation amplitude of the membrane potential to a “ZAP” current stimulation increases, as shown by the difference between the maximal and minimal values of the membrane potential in Figure 11A ($I_{app}=-0.05\mu \text{A}/\text{c}{\text{m}}^{2}$) and Figure 11B ($I_{app}=0.08\mu \text{A}/\text{c}{\text{m}}^{2}$). The increase of the oscillation amplitude of the membrane potential leads to the increase of the maximal impedance with increasing *g*_*h*_. Such a result is consistent with bifurcation theory wherein as a stable dynamical behavior approaches the bifurcation point, its capability of resisting disturbance such as regular stimulus becomes weaker. For the stable node or stable focus which should keep unchanged and remain at a fixed level, the weakness of the capability of resisting disturbance with increasing *g*_*h*_, i.e., approaching the bifurcation point, is characterized by the increase of the oscillation amplitude of the membrane potential to the “ZAP” current stimulus.

Last, complex or “strange” phenomenon related to the subthreshold resonance appears near the bifurcation point. (1) In the small parameter region of the resting state or focus down-right to the BT point or SNHO point, not “typical”-resonance, but non-resonance or “weak”-resonance (Figure 7), appears for the stable focus, which is “strange” because a “typical”-resonance should appear. The dynamical mechanism is very complex and may be from the narrow region of focus and the close distance from the codimension-2 bifurcation point BT with double zero eigenvalues. Such a complex dynamical behavior is similar to those simulated in the parameter region near a special equilibrium with double zero eigenvalues in a linear model (Rotstein, 2013; Rotstein and Nadim, 2014). The dynamical mechanism underlying the nonlinear system used in the present paper and the difference to the linear system should be studied in future. (2) The “weak”-resonance appears for the stable node within the neighborhood of the transition curve between the stable node and focus, which is also “strange” due to a non-resonance should appear for a stable node. Such a result also shows that the complex behaviors appear near the bifurcation/transition point. Except for the bifurcation/transition point, the external stimulus signal, the “ZAP” current, may be an important factor to induce the “weak”-resonance for the stable node. (3) There is complex phenomenon in the very narrow region of the coexistence behavior. We do not address the phenomenon because the parameter region is very narrow. The complex dynamics within these three narrow parameter regions (Figure 9D) near the bifurcation/transition point should be further studied in future. Except for the three narrow regions, the “typical”-resonance and non-resonance appear in the parameter region of the stable focus and the stable node far away from the bifurcation/transition point, respectively.

#### Dynamical mechanism of *I*_*h*_ induced more spike-timing precision

**Figure 12:**
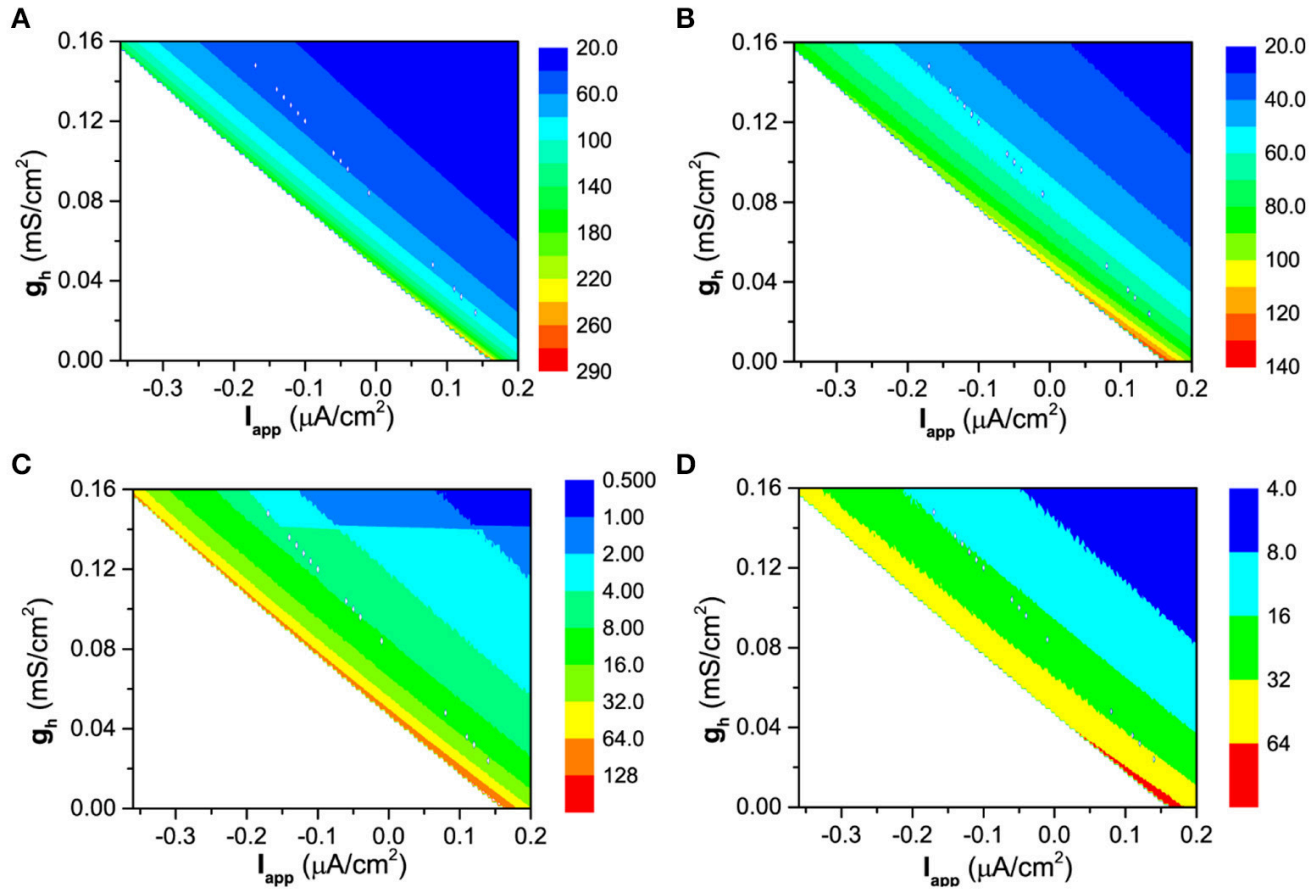
The dependence of the mean value and standard deviation of ISIs on both *I*_*app*_ and *g*_*h*_ at different levels of noise intensity *D*. Mean value of ISIs: **(A)** *D* = 0.2μA/cm^2^; **(B)** *D* = 0.6μA/cm^2^; Standard deviation of ISIs: **(C)** *D* = 0.2μA/cm^2^; **(D)** *D* = 0.6μA/cm^2^.

**Figure 13:**
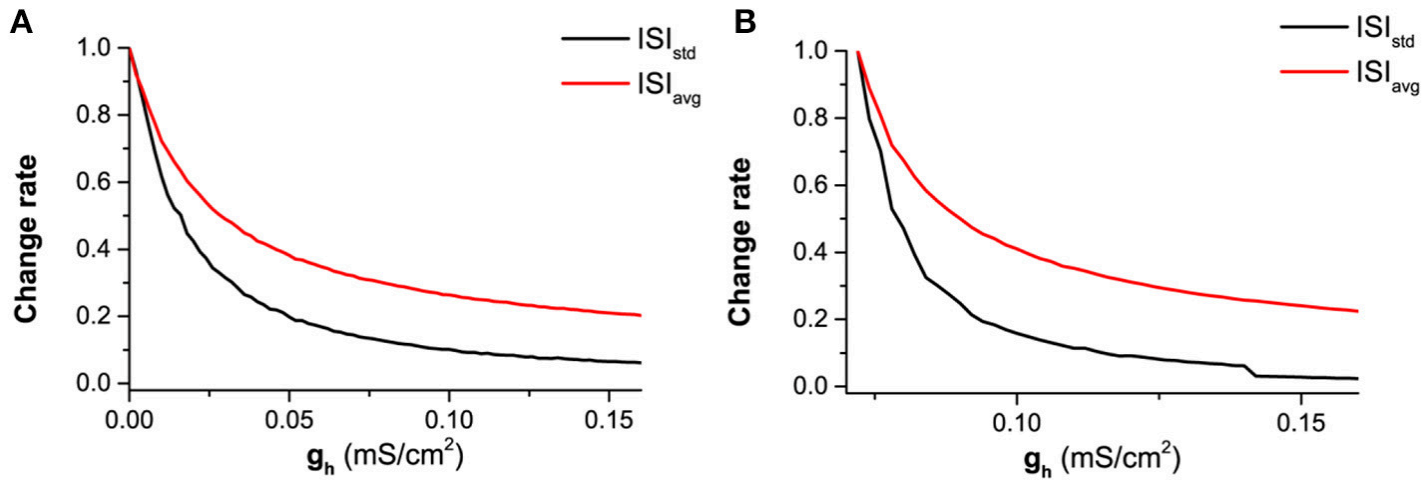
The decrease of normalized mean value (red) and standard deviation (black) of ISIs with increasing *g*_*h*_ at different levels of *I*_*app*_ and noise intensity *D*. **(A)** *I*_*app*_ = 0.17 μA/cm^2^ and *D* = 0.6μA/cm^2^; **(B)** *I*_*app*_ = −0.08 μA/cm^2^ and *D* = 0.2μA/cm^2^. ISI_std_ (black) and ISI_avg_ (red) represent the standard deviation and mean value of ISIs.

The more spike-timing precision induced by *I*_*h*_ can also well be understood with the bifurcation theory. For the firing behavior, the increase of *g*_*h*_ means that the firing becomes farther away from the bifurcation point. According to the bifurcation theory, as a stable dynamical behavior becomes farther away from the bifurcation point, its capability of resisting disturbance such as noise becomes stronger (Izhikevich, 2000, 2007; Tateno et al., 2004). For a neuronal firing, the enhancement of the capability of resisting disturbance is characterized by the further decrease of the standard deviation (*STD*) of ISIs than the mean value of ISIs, with increasing *g*_*h*_, as shown in Figures 12, 13, which leads to the decrease of *CV* of ISIs, i.e., the enhancement of the spike-timing precise.

The faster decrease of *STD* than the mean value can be found from Figure 12. For *D* = 0.2μA/cm^2^, the mean value decreases from 290 ms to 20 ms, as shown in Figure 12A, and the *STD* decreases from 128 ms to 0.5 ms, as shown in Figure 12B. 290/20 (14.5) is much smaller than 128/0.5(256). For *D* = 0.6μA/cm^2^, the mean value decreases from 140ms to 20ms, as depicted in Figure 12C, and the *STD* decreases from 64ms to 4ms, as shown in Figure 12D. 140/20 (7) is smaller than 64/4(16). The results are similar in a large noise intensity (0 < *D* < 1.0μA/cm^2^).

The faster decrease of the *STD* than the mean value can also be found from two representatives shown in Figure 13. If the mean value for different *g*_*h*_ values are normalized by the largest value corresponding to the smallest *g*_*h*_, the decrease of the normalized values with increasing *g*_*h*_ is shown by the red line in Figure 13A (*I*_*app*_ = 0.17 μA/cm^2^ and *D* = 0.6μA/cm^2^) and Figure 13B (*I*_*app*_ = −0.08 μA/cm^2^ and *D* = 0.2μA/cm^2^). Similarly, the decrease of the normalized *STD* with increasing *g*_*h*_ is depicted by the black line in Figures 13A,B). For both representatives, the *STD* (black) decreases faster than the mean value (red).

The results show that the enhancement of the spike-timing precision is mainly caused by the fast decrease of standard deviation of ISIs with increasing *g*_*h*_, which obeys the bifurcation theory wherein a stable dynamical behavior far away from the bifurcation point exhibits stronger capability of resisting disturbance such as noise.

#### Dynamical mechanism of *I*_*h*_ induced rebound (spike)

The voltage sag is mainly induced by the hyperpolarization activation of *I*_*h*_, which can be found from the 2nd−4th panels of Figures 2A–C. At the initial phase of the hyperpolarization current, *I*_*h*_ increases with increasing time, which leads to the increase of the membrane potential, i.e., the voltage sag. If the strength of the hyperpolarization current is stronger, *I*_*h*_ becomes higher. Correspondingly, the voltage sag becomes stronger. If *g*_*h*_ is larger, *I*_*h*_ is higher, which also leads to the stronger voltage sag. If there is no *I*_*h*_, no voltage sag appears.

The rebound (spike) is mainly induced by both *I*_*h*_ and Na^+^ current, as shown by the 2nd to 4th panels of Figure 2. At the termination time of the hyperpolarization current, *I*_*h*_ is much higher than that of the resting state, which leads to the depolarization of the membrane potential. The depolarization of the membrane potential induces the decrease of *I*_*h*_ and the increase of Na^+^ current which plays the dominant role and leads to further depolarization of the membrane potential. The larger the *g*_*h*_, the higher the *I*_*h*_; the larger the strength of the hyperpolarization square current, the higher the *I*_*h*_. When *g*_*h*_ and/or the strength of the hyperpolarization current are lower, *I*_*h*_ is lower and the depolarization is not strong enough to induce a spike, which corresponds to the rebound phenomenon. When *g*_*h*_ and/or the strength of the hyperpolarization current are larger, *I*_*h*_ is higher and the depolarization is strong enough to induce a spike, which corresponds to the rebound spike phenomenon.

The rebound (spike) becomes strong with increasing *g*_*h*_, which can also be well understood with the bifurcation theory. According to the bifurcation theory (Izhikevich, 2000, 2007), as a stable dynamical behavior approaches the bifurcation point, its capability of resisting disturbance such as regular stimulus becomes weaker. For the resting state, the increase of *g*_*h*_ means that it approaches the bifurcation point, and the weakness of the capability of resisting disturbance such as the hyperpolarization square current is characterized by the stronger rebound (spike).

For the resting state for other *I*_*app*_ values, the results are similar to those obtained with *I*_*app*_ = −0.05 μA/cm^2^. If there is no *I*_*h*_, no rebound (spike) is induced.

## Discussion

Experimental studies found that *I*_*h*_ contributes to the voltage sag and rebound (spike) to the inhibitory square current, resonance to sinusoidal current input, and more spike-timing precision (Zemankovics et al., 2010; Engbers et al., 2011; Gastrein et al., 2011; Pavlov et al., 2011; Borel et al., 2013; Gonzalez et al., 2015), which are rarely explained theoretically. In the present paper, with increasing *g*_*h*_, stronger voltage sag/rebound (spike) and resonance, and more spike-timing precision are simulated in a theoretical model. In addition, the enhancement of the membrane potential for the resting state, the increase of firing frequency for the firing behavior, and the decrease of the firing threshold for *I*_*app*_ induced by *I*_*h*_ are also simulated in the model. All results closely match those of the previous experiment, which shows that the theoretical model is suitable to investigate the dynamics of neurons with *I*_*h*_.

With help of the complex bifurcations and the distribution of the stable behaviors in (*I*_*app*_, *g*_*h*_) plane, the dynamical mechanism that can well interpret the experimental observations is provided. With increasing *g*_*h*_, the four stable behaviors are stable node, stable focus, coexistence behaviors, and the firing, which are separated by the bifurcations. For the firing behavior, the increase of *g*_*h*_ means that the firing becomes far away from the bifurcation point. According to the bifurcation theory, as a stable dynamical behavior becomes far away from the bifurcation point, its capability of resisting disturbance such as noise becomes strong. It is the cause that *CV* and standard deviation of ISIs become lower with increasing *g*_*h*_, i.e., the enhancement of the spike-timing precision. For the resting state, increasing *g*_*h*_ means that the resting state approaches to the bifurcation point. As a stable dynamical behavior approaches to the bifurcation point, its capability of resisting disturbance such as hyperpolarization stimulus becomes strong. It is the cause that rebound (spike) becomes strong with increasing *g*_*h*_.

Compared with the rebound (spike) and spike-timing precision, the dynamical mechanism for the subthreshold membrane resonance is very complex. It can be concluded that the “typical”-resonance appears in the parameter region of the stable focus far away from the bifurcation point, and non-resonance phenomenon appears in the parameter region of the stable node far away from the bifurcation point. However, complex or “strange” dynamics appear in the parameter region near the bifurcation/transition point. For example, the non-resonance phenomenon appears in the parameter region of the stable focus near a codimmenion-2 bifurcation point with double zero eigenvalues, the BT point, and “weak”-resonance appears in the parameter region of the stable node near the transition point between the stable focus and node. These results are similar to those simulated near the special point with double zero eigenvalues in a linear system (Rotstein, 2013; Rotstein and Nadim, 2014). In the experiment (Zemankovics et al., 2010; Gastrein et al., 2011; Pavlov et al., 2011; Borel et al., 2013; Gonzalez et al., 2015), the “typical”-resonance appears in the presence of *I*_*h*_ and non-resonance appears when *I*_*h*_ is blocked. According to the results of the present paper, the behavior is stable focus for the “typical”-resonance and stable node for the non-resonance phenomenon, which shows that *I*_*h*_ induces a transition from the stable node to stable focus. In nonlinear dynamics, the stable focus related to Hopf bifurcation and the stable node associated with SN/SNIC bifurcation. Therefore, the transition point between Hopf bifurcation and SN/SNIC bifurcation, the BT point, plays important roles in the determination of the dynamics of the neuronal system with *I*_*h*_. The underlying mechanism for the complex or “strange” dynamics, which may be related to the BT point (Izhikevich, 2000, 2007; Zhao and Gu, 2017), should be studied in future.

With help of the results of the present paper, many other physiological activities observed in the experiment, such as the phase response curve (Ermentrout, 1996; Smeal et al., 2010), synchronization (Hansel et al., 1995; Ermentrout, 1996; Fink et al., 2011; Tikidji-Hamburyan et al., 2015), spike initiating dynamics (Prescott et al., 2008), and neural coding efficiency (Yi et al., 2015), can be further understood. For example, neuron with *I*_*h*_ exhibits resonance at a certain frequency and firing with a fixed frequency, which contributes to the formation of synchronized oscillatory (Zemankovics et al., 2010; Stark et al., 2013). It is also the cause that the synchronized oscillatory appears in the hippocampal pyramidal neurons and stratum oriens interneurons (Varga et al., 2008; Colgin, 2013). In addition, *I*_*h*_ can induce bistability or coexistence of the resting state and firing (Figures 6B,C). The parameter region of the bistability or coexistence of the resting state and firing, i.e., the parameter region lying between the SN/SNIC curve (black dashed line) and BHom curve (blue line), becomes wide as the conductance of *I*_*h*_ (*g*_*h*_) increases (Figure 9), which is consistent with the previous experimental observation wherein *I*_*h*_ can enlarge the region of bistability or coexistence in the Purkinje cell (Engbers et al., 2013), which is a potential mechanism for the short-term memory (Durstewitz et al., 2000; Loewenstein et al., 2005; Engbers et al., 2013).

## Author contributions

ZZ, LL, and HG: conceived and designed the work; ZZ and LL: performed the simulations; ZZ, LL, and HG: analyzed and interpreted the data; ZZ and HG: wrote the paper.

### Conflict of interest statement

The authors declare that the research was conducted in the absence of any commercial or financial relationships that could be construed as a potential conflict of interest.
